# Quantifying physiological trait variation with automated hyperspectral imaging in rice

**DOI:** 10.3389/fpls.2023.1229161

**Published:** 2023-09-20

**Authors:** To-Chia Ting, Augusto C. M. Souza, Rachel K. Imel, Carmela R. Guadagno, Chris Hoagland, Yang Yang, Diane R. Wang

**Affiliations:** ^1^ Agronomy Department, Purdue University, West Lafayette, IN, United States; ^2^ Institute for Plant Sciences, Purdue University, West Lafayette, IN, United States; ^3^ Botany Department, University of Wyoming, Laramie, WY, United States

**Keywords:** *Oryza sativa*, genetic diversity, growth traits, high-throughput phenotyping, nitrogen

## Abstract

Advancements in hyperspectral imaging (HSI) together with the establishment of dedicated plant phenotyping facilities worldwide have enabled high-throughput collection of plant spectral images with the aim of inferring target phenotypes. Here, we test the utility of HSI-derived canopy data, which were collected as part of an automated plant phenotyping system, to predict physiological traits in cultivated Asian rice (*Oryza sativa*). We evaluated 23 genetically diverse rice accessions from two subpopulations under two contrasting nitrogen conditions and measured 14 leaf- and canopy-level parameters to serve as ground-reference observations. HSI-derived data were used to (1) classify treatment groups across multiple vegetative stages using support vector machines (≥ 83% accuracy) and (2) predict leaf-level nitrogen content (N, %, *n=88*) and carbon to nitrogen ratio (C:N, *n=88*) with Partial Least Squares Regression (PLSR) following RReliefF wavelength selection (validation: *R* ^2 = ^0.797 and RMSEP = 0.264 for N; *R* ^2 = ^0.592 and RMSEP = 1.688 for C:N). Results demonstrated that models developed using training data from one rice subpopulation were able to predict N and C:N in the other subpopulation, while models trained on a single treatment group were not able to predict samples from the other treatment. Finally, optimization of PLSR-RReliefF hyperparameters showed that 300-400 wavelengths generally yielded the best model performance with a minimum calibration sample size of 62. Results support the use of canopy-level hyperspectral imaging data to estimate leaf-level N and C:N across diverse rice, and this work highlights the importance of considering calibration set design prior to data collection as well as hyperparameter optimization for model development in future studies.

## 1 Introduction

Variation in plant traits reflect differences in genetics, the environment, and their interactions integrated over time (Allard and Bradshaw, 1964). Understanding these relationships could provide mechanistically-based insights into breeding climate-resilient crops, however, conventional methods to measure plant traits can be destructive, may be time-consuming, and often require specific technical skills that vary across methods. These characteristics make such approaches challenging to scale across the large, and often diverse, germplasm panels that are relevant to geneticists and breeders. Deriving trait data from imaging as a systematic means of non-destructive, high-throughput phenotyping has therefore emerged as an important research area for the plant research community over the past decade (Yang et al., 2014; Mir et al., 2019; Yang et al., 2020). For example, morphometric traits (e.g., plant surface area and plant height) are now routinely extracted from Red-Green-Blue (RGB) images using established analysis pipelines (Yang et al., 2014; Gehan et al., 2017; Berry et al., 2018; Kim et al., 2020). In contrast, analogous pipelines for predicting physiological responses and/or biochemical traits from image-derived data have not been well-established (Pasala and Bb, 2020; Yang et al., 2020) and represents a significant gap for plant research communities.

Out of the various types of imaging technologies available to plant researchers, those with hyperspectral sensors have shown the greatest promise for estimating physiological and biochemical traits in plants. These imaging systems are made up of a light source, objective lenses, an imaging spectrograph, hyperspectral sensor(s), and a computer. Results are stored as quantitative electrical signals derived from a vast number of images, each corresponding to the reflectance value – the ratio of reflected radiant flux to the incident flux – of wavelengths ranging between 400 to 2500 nm (Sarić et al., 2022). As early as the 1970s, plant scientists have documented the relationship between leaf traits (e.g., thickness, water content, presence of wax and hairs, and age) and hyperspectral reflectance (Gausman and Allen, 1973; Grant, 1987). General signatures observed for reflectance of plant tissue have also been linked to function and composition. For example, low reflectance in the visible light region (VIS, 480 – 510 nm and 640 – 670 nm) is due to the absorption of light by photosynthetic pigments, reflectance in the near infrared region (NIR, 700 – 1100 nm) is influenced by the arrangement of mesophyll tissues of leaves (Rouse et al., 1974), and the two troughs observed in the short–wave infrared region (SWIR, 1000 – 2500 nm) are affected by plant cell water content (Cotrozzi et al., 2020).

Previous studies have related HSI-derived data to both quantitative and qualitative crop responses to abiotic factors. From controlled-environment phenotyping facilities, these include studies on maize response to different watering regimes (Ge et al., 2016; Asaari et al., 2019; Mertens et al., 2021), quantification of macronutrients in both maize and soybean Pandey et al. (2017), and generation of nitrogen distribution maps at the whole-plant level in wheat Bruning et al. (2019). Cultivated Asian rice (*Oryza sativa*), consumed directly by more than half of the world’s population (Muthayya et al., 2014), has also been characterized for its spectral features under field and controlled environment conditions (Arias et al., 2021). For example, Din et al. (2017) reported that leaf area index during the vegetative stage could be estimated from Vegetation Indices (VIs) derived from hyperspectral data using a single *japonica* variety. These experiments were carried out in the field using a spectroradiometer to collect canopy-level data. Spectral data have also been used to detect common leaf diseases across four varieties of rice grown in greenhouse conditions (Feng et al., 2021). For that study, leaves were first sampled destructively and subsequently placed in a hyperspectral imaging system. Yu et al. (2020) developed leaf-level nitrogen content models from spectral data for one *japonica* variety and one *indica* variety under field conditions. Their canopy-level HSI data were collected with a spectroradiometer, and specific VIs were used as model input. While results were promising in each of these rice studies, previous work focused on small numbers of accessions; the potential scalability and general utility of hyperspectral imaging data across more diverse germplasm remain to be tested.

To help address this gap, the current study evaluates 23 genetically diverse *O. sativa* accessions from two divergent subpopulations grown under two nitrogen levels in a phenotyping facility equipped with automated hyperspectral imaging. To establish potential relationships between physiologial traits and HSI-derived data, the automated imaging is complemented by a suite of ground-reference observations. While numerous approaches exist that could be considered for analyzing these high dimensional and multi-collinear hyperspectral data Mir et al. (2019); Mishra et al. (2020); Arias et al. (2021), we choose to leverage support vector machines (SVMs) for classification, as they have been recognized as an effective image classification algorithm Noble (2006); Gewali et al. (2018), and Partial Least Squares Regression (PLSR) as a computationally-tractable method for trait prediction Burnett et al. (2021). In this study, application of SVM and PLSR follow Principal Components Analysis (PCA) and the RRefliefF algorithm, respectively, to retain only the most critical information from the original hyperspectral data. PCA is a classic example of an unsupervised method to reduce data dimensionality Gewali et al. (2018); Yu et al. (2020), while the RRefliefF algorithm is a supervised, ranking-based method that calculates an importance score of each wavelength by considering the similarity and dissimilarity between wavelengths Ren et al. (2020).

The overall goal of our study is to test the utility of data derived from an automated hyperspectral imaging system as surrogates for physiological traits across genetically diverse rice. Specific objectives are to (1) assess whether HSI-derived data can classify subpopulation and treatment groupings across time, (2) understand which types of plant traits have the most potential to be predicted, (3) evaluate whether models developed using a single subpopulation or treatment grouping can be used to predict values in the other, and (4) quantify the effects of hyperparameter combinations on predictions by the RReliefF-PLSR framework.

## 2 Materials and methods

### 2.1 Plant materials

A set of 23 bio-geographically diverse accessions from two publicly-available, purified germplasm collections, the Rice Diversity Panel (RDP) 1 and RDP 2 (McCouch et al., 2016), were evaluated for this study. These 23 lines encompassed 15 *indica* and eight *tropical japonica* accessions that originated from 17 countries. (**Figure S1**). To limit potential confounding effects of development on target traits, these accessions were selected out of the tropically-adapted and phenologically-similar RDP subset screened by Wang et al. (2016b) (**Table S1**). Seeds were obtained from the USDA-ARS, Dale Bumpers National Rice Research Center, Stuttgart, Arkansas, Genetic Stocks Oryza Collection (https://www.ars.usda.gov/GSOR).

### 2.2 Growth conditions

The selected diversity panel was raised at the Ag Alumni Seed Phenotyping Facility (AAPF), a controlled environment high-throughput plant phenotyping facility at Purdue University (West Lafayette, Indiana, U.S.A.) for 94 days during the Summer and Fall of 2020 (**Figure S2**). The facility is made up of a fully automated growth chamber (Conviron^®^, Winnipeg, Canada) and weight-based irrigation system (Bosman Van Zaal, Aalsmeer, The Netherlands). A virtual tour of the facility may be found at https://ag.purdue.edu/aapf/virtual-tour.html. Three conveyer belts with 32 positions per belt were allocated to this study. Of the 96 total positions, two were designated as “purge pots,” i.e., pots into which the system flushes solutions in between changing fertigation/irrigation regimes. The remaining 94 positions were occupied by 22 genotypes x 2 replicates x 2 nutrient levels and one final genotype (cv. Cybonnet) x 3 replicates x 2 nutrient levels (described below). Having two or more replicates per genotype allowed us to calculate accession-level means for exploring data structure, i.e., subpopulation or treatment. The temperature setpoint in the chamber was 26/22°C day/night, relative humidity at 60%, and photosynthetically active radiation (PAR) levels were recorded between 550-600 *µmol* photons *m* ^−2^ *s* ^−1^.

The environment was additionally tracked by affixing Lascar EL-USB-2-LCD Data Loggers to seven randomly selected pots at the sowing time, which recorded temperature and relative humidity every 10 minutes (**Figure S3A**). Average temperature and humidity from the loggers across the experimental period were 29.48 ± 0.22/23.34 ± 0.09°C day/night, relative humidity at 64.62 ± 0.69%. Lighting conditions followed long day (14 h day/10 h night) scheduling due to the need to accommodate experiments on other species in the same facility: lights turned on daily at 0600 h and turned off at 2000 h. Two seeds were sown per pot (6L in volume) in horticultural substrate, which was made up of a mixture of Profile Porous Ceramic Greens Grade and Berger BM6 All Purpose at a one-to-one ratio by volume. Plants were hand-watered until 10 days after sowing (DAS), at which point the seedlings were thinned and a weight-based automated irrigation was initiated. The experiment was designed with two nutrient treatment levels: high (300 ppm nitrogen, N1) and low (50 ppm nitrogen, N2). The fertigation solution was created by mixing Peters Excel 15-5-15 Cal Mag Special in reverse osmosis (RO) water. Each morning prior to chamber lights turning on, plants were irrigated to a preset weight with RO water. This target weight was increased by about 1.15 times during the experiment to account for the increase in transpirational demand of the growing plants (**Figure S3B**). RO water irrigation occurred every day except on scheduled days when a fixed volume of fertigation solution (either high or low concentration, depending on the treatment) was applied instead of RO water. This RO water irrigation and fertigation regime was designed so that each plant should theoretically receive enough water to meet individual transpirational demands while also receiving the fertilizer amount prescribed by their treatment.

### 2.3 Imaging and image processing

Plants were imaged approximately three times per week beginning on 20 DAS and continuing until the end of the experiment using the automated imaging booth in AAPF. This temporal frequency enabled us to average HSI data within each week to reduce the influence of noisy spectra. During each imaging event, one side-view and one top-view images were acquired, though the current study analyzed side-view data only as top-view images after 46 DAS were unavailable due to a camera malfunction. The HSI cameras used a scanning range that encompassed the VIS to NIR region (VNIR; 400 – 1000 nm, MSV 500 VNIR Spectral Camera, Middleton, Spectral Vision, WI, U.S.A.) and the SWIR region (Specim, Oulu, Finland); thus, generation two hypercubes of data image. White and dark reference tests were conducted for each hyperspectral cameras (SVNIR and SWIR) for post-processing images, where the relative light reflectance was estimated for each wavelength. This step was based on the work by Zhang et al. (2019). For the white reference, two panels of known material and spectral signature was scanned with all lights inside the imaging chamber on. The dark reference test was conducted with the lens shutter closed and no lights. The relative light reflectance (%) for each plant was calculated based on the normalized difference between these tests, as seen in Equation 1.


(1) $$reflectance\;(\%)=100\times \frac{Plant-Dark}{White-Dark}$$

where,

Plant – the raw digital number measured for the rice plants

Dark - the raw digital number measured during the dark reference tests

White - the raw digital number measured during the white reference tests

For the VNIR hypercube, the rice plants were segmented out of the background using the typical attenuation between Red Edge wavelengths reflectance. The SWIR hypercube segmentation was done using the SURF image registration (Bay et al., 2008) to map the rice plants from the segmented VNIR hypercube (fixed image) using the VNIR-segmented rice plant as the fixed image. After the segmentation, the average light reflectance was calculated and stored in a spreadsheet. Hyperspectral images were processed by using a proprietary processing script in MATLAB (MATLAB, 2018).

### 2.4 Growth and physiological measurements

**Figure 1:**
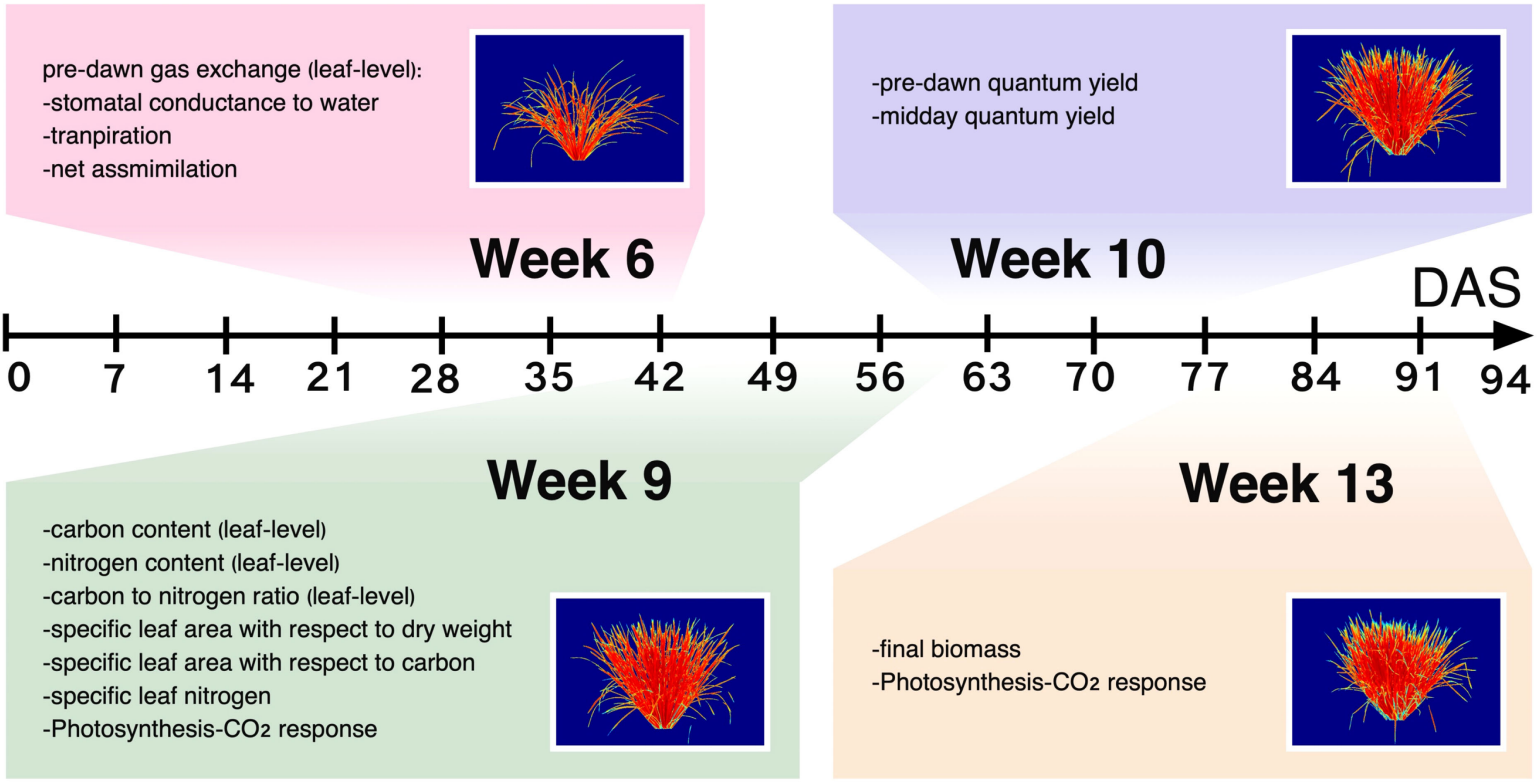
Overview of experimental data collection. Fourteen physiological traits were collected at different timepoints over the course of plant development. Hyperspectral imaging was carried out several times per week and imaging events that occurred approximately within the same weeks as phenotyping campaigns were averaged for analyses. The false-colored images in boxes show the same individual rice plant over the four measurement weeks.

Pre-dawn gas exchange measurements were taken in the facility growth chamber during early vegetative growth between 0430-0530 h prior to chamber lights turning on using LI-6800 Portable Photosynthesis System (LI-COR, Lincoln, NE, USA) (**Table 1**). Leaf-level net assimilation (A, *µmolm* ^−2^ *s* ^−1^), stomatal conductance to water vapor (gsw, *molm* ^−2^ *s* ^−1^), and nighttime transpiration (E, *molm* ^−2^ *s* ^−1^) were extracted from the measurements. Environmental conditions in the cuvette matched ambient conditions in the growth chamber: reference *CO*_2_, 415 *µmolmol* ^−1^; vapor pressure deficit,< 1.5*kPa* (average *V PD* = 1.09*kPa*); PAR, 0 *µmol* photons *m* ^−2^ *s* ^−1^; and leaf temperature was 26.87 ± 0.016 °C (mean ± SE). As rice leaves are generally too narrow to cover the entire cuvette, leaf width was first measured with a digital caliper prior to each measurement in order to adjust gas exchange measurements to the observed leaf area. A/Ci curves were collected in the facility growth chamber using both LI-6800 and LI-6400XT Portable Photosynthesis Systems (LI-COR, Lincoln, NE, USA) during 59-63 DAS and 87-91 DAS (mid-tillering and late vegetative stages, respectively) with constant PAR of 1200 *µmol* photons *m* ^−2^ *s* ^−1^ and reference *CO*_2_ concentrations were set in the following order: 415, 300, 200, 100, 50, 10, 415, 415, 600, 800, 100, 1200, 415 *µmolmol* ^−1^. Light response curves were initially conducted on randomly selected plants to determine the PAR level for running A/Ci curves. A/Ci curves were collected between 1000 and 1400 h on the youngest fully expanded leaf as indicated by the emergence of the leaf collar. Leaf temperatures were between 25 and 27 °C and relative humidity was maintained between 50-70%. After A/Ci curves were run during 59-63 DAS, the leaf used for each curve was excised and its area measured using a leaf area meter (LAI-2200C; LI-COR, Lincoln, NE, USA). Leaves were oven dried for at least five days at 65 °C and their dry weights recorded. They were then finely ground using a mortar and pestle and subsequently analyzed for carbon (*C*, %) and nitrogen (*N*, %) content using the FlashEA^®^ 1112 Nitrogen and Carbon Analyzer for Soils, Sediments and Filters (Thermo Scientific, CE Elantech, Lakewood, NJ) with two replicates *per* leaf (30 mg per replicate). The equipment was operated according to the flash dynamic combustion method, and resultant signals were translated into the percentage of carbon and nitrogen by the Eager 300 software. During 67-91 DAS, chlorophyll a fluorescence was measured at predawn (0430-0530 h) and midday (1000-1400 h) conditions with a hand-held fluorometer (Fluorpen FP110, Photon System Instruments, Drasov, Czech Republic) on the youngest, fully expanded leaf per plant on three different areas of the leaf blade to minimize possible variation in the efficiency of PSII due to the spatial response to sudden environmental changes in monocotyledons (Oberhuber et al., 1993): the basal one-third, the middle one-third, and the distal one-third. Measurements of Fv/Fm, the maximum efficiency of PSII in dark acclimated leaves, were taken according to Murchie and Lawson (2013). The measuring light of the FluorPen was set at approximately 900 *µmol* photons *m* ^−2^ *s* ^−1^ throughout the experiment. Then, we applied a saturation pulse at approximately 1500 *µmol* photons *m* ^−2^ *s* ^−1^to obtain Fv/Fm (pQY), whereas those taken at midday were the maximum efficiency of PSII in light conditions, Fv’/Fm’(mQY) (Henriques, 2009). Total above-ground biomass from all plants were harvested at the end of the experiment on 94 DAS, oven-dried at 65 °C for at least five days, and their dry weights recorded. **Table S2** summarizes the relationship between the timing for physiological trait measurements and the imaging dates and **Figure 1** provides an overview of the experiment.

**Table 1:** Overview of growth and physiology measurements.

Abbreviation	Units	Description
*A*	*µmolm* ^−2^ *s* ^−1^	pre-dawn leaf-level net assimilation
*C*	%	carbon
*C*: *N* (or CN in Figures)	–	leaf-level carbon to nitrogen ratio
*E*	*molm* ^−2^ *s* ^−1^	pre-dawn leaf-level transpiration
*FB*	g	final biomass
*gsw*	*molm* ^−2^ *s* ^−1^	pre-dawn leaf-level stomatal conductance to water
*Jmax*	*µmolm* ^−2^ *s* ^−1^	light-saturated potential rate of electron transport
*log*(*E*)	–	log transformed pre-dawn leaf-level transpiration
*log*(*gsw*)	–	log transformed pre-dawn leaf-level stomatal conductance to water
*log*(*SLN*)	–	log(specific leaf nitrogen)
*mQY*	–	midday quantum yield
*N*	%	nitrogen
*normmQY*	–	normalized midday quantum yield
*normpQY*	–	normalized pre-dawn quantum yield
*pQY*	–	pre-dawn quantum yield
*SLA*	*cm* ^2^ *g* ^−1^ *dw*	specific leaf area with respect to dry weight
*SLA_C_*	*cm* ^2^ *mg* ^−1^ *C*	specific leaf area with respect to carbon
*SLN*	*mgNcm* ^−2^	specific leaf nitrogen
*Vcmax*	*µmolm* ^−2^ *s* ^−1^	the maximum carboxylation rate limited by Rubisco

### 2.5 Data analysis

Data were formatted and analyzed in R 4.1.1 (R Core Team, 2021) with packages dplyr (Wickham et al., 2023), tidyverse (Wickham et al., 2019) and reshape2 (Wickham, 2007). Plots were generated using the package ggplot2 (Wickham, 2016) or in base R environment. The code for each physiological trait prediction model can be accessed through GitHub (https://github.com/To-Chia/rice_imaging_ms).

#### 2.5.1 Physiological trait analysis

From the ground-reference observations, we derived specific leaf area (*SLA*, *cm* ^2^ *g* ^−1^), CN ratio (*C*: *N*), specific leaf area with respect to carbon (*SLA_C_* (*cm* ^2^ *mg* ^−1^(*C*)) and specific leaf nitrogen (*SLN*, *mg*(*N*)*cm* ^−2^). The summary statistics may be found in **Table S3**. *E* and *gsw* had right-skewed distributions and thus were log-transformed (denoted as *log*(*E*) and *log*(*gsw*), respectively), prior to conducting the correlation tests and a PCA. Pearson’s correlation coefficient was calculated for all pairwise combinations of the physiological traits and plotted with package corrplot (Wei and Simko, 2021) (**Figure S4A**). Data for *SLA*, *C* and *N* on six rice plants were unavailable and were mean-imputed before the pairwise correlation was calculated. The same methods were applied to *C*: *N*, *SLA_C_*, and *SLN*. A/Ci curves were first assessed for quality control (QC) using the PEcAn.photosynthesis package (https://pecanproject.github.io/modules/photosynthesis/docs/articles/ResponseCurves.html). For the 89 curves that passed QC, *V_cmax_* and *J_max_* were estimated using the R plantecophys package (Duursma, 2015). PCA based on correlation matrix was conducted on accession-mean data using the prcomp function in base R (R Core Team, 2021). For unavailable data, their replicates were used to represent the accessionmean value. Agglomerative hierarchical clustering was performed on the Euclidean distance matrix from accession-mean data. Clustering was based on the average linkage method and plotted with package dendextend (Galili, 2015).

#### 2.5.2 Hyperspectral imaging data

To obtain stable signals for modeling, imaging events that occurred approximately within the same weeks were averaged (**Table S2**; **Figure 1**). Averaged datasets are hereafter referred to as Week 6, 9, 10 and 13 HSI data. PCA with variance-covariance matrix was conducted on weekly accession-mean HSI data. From the results of PCA, wavelengths that had the top ten loadings of the PCs that cumulatively accounted for *>* 90% of total variance were selected and termed as *W_SVM_*.

#### 2.5.3 Signal variation in HSI data through time

SVMs were trained to quantify the prediction accuracy of treatment groupings from *W_SVM_* using the package e1071 (Meyer et al., 2021). First, the classifier of the training week was built with *W_SVM_*. Then, predictions made on the evaluation week were achieved by selecting *W_SVM_* in the training week dataset from the evaluation week. We tested two kernels, the radial basis function (RBF) kernel and the linear kernel. Grid-search of the parameters for both kernels were conducted with 23-fold cross validation. Both rough and fine grid-searches were conducted. In the rough grid-search of RBF kernel, parameters *cost* and *gamma* were evaluated within the range of $\left(2^{-5},2^{15}\right),\left(2^{-15},2^{23}\right)$ , respectively, both with an interval of 2^2^.

The best parameters resulting from the rough grid-search, +/- 2^0.5^, set the range of the subsequent fine-grid search with an interval of 2^0.1^. When the best parameters from the rough grid-search fell on the boundary, ranges for the fine grid-search used the best value plus or minus 2 within the specified ranges. Parameters found in the fine grid-search were adopted only if model accuracy was higher in the fine grid-search than the rough grid-search; in some cases, accuracies were the same. The parameter, *cost*, which was the only parameter in the linear kernel, was determined with the same method as *cost* in the RBF kernel.

#### 2.5.4 Prediction of physiological traits with HSI data

We used the RReliefF algorithm implemented by the CORElearn package (Robnik-Sikonjǎ and Kononenko, 2003) to select relevant wavelengths before utilizing partial least squares regression (PLSR) for predictions (**Figure S5**). RReliefF algorithms are derived from Relief, developed by Kira and Rendell (1992). We chose RReliefF to filter HSI data as it takes conditional dependencies between variables into account (Robnik-Sikonjǎ and Kononenko, 2003). Wavelengths selected from RReliefF were termed as *W_PLSR_*. The estimator applied in the current study was RReliefF expRank, and the number of iterations was determined by the number of calibration samples multiplied by 100. Note that calibration in this study refers to model training in the context of machine learning while model validation is equivalent to model testing. For PLSR modeling, the R package, pls (Liland et al., 2021), was used, and the procedure for building the models was adapted from Burnett et al. (2021). Specifically, models were fit using the classical orthogonal scores algorithm. Eighty percent of the full dataset was used for calibration and the remaining was for model validation. Sampling was carried out with the criterion that each treatment level contributed equally to the datasets (*i.e.*, the full-data was grouped by treatments prior to sampling). Calibration datasets were used to determine the number of components in the final models by selecting the lowest root mean square error of prediction (*RMSEP*, Equation 2) in leave-one-out (LOO) cross-validation. The coefficient of determination (*R* ^2^, Equation 3), *RMSEP* and normalized root mean square of error in predictions (%*RMSEP*, Equation 4) served as model evaluation metrics.


(2) $$RMSEP=\sqrt{\frac{\sum_{i=1}^{n}\left(y_{i}-{\hat{y}}_{i}\right)2}{n}}$$

(3) $$R^{2}=1-\frac{\sum_{i=1}^{n}{\left(y_{i}-{\hat{y}}_{i}\right)}^{2}}{\sum_{i=1}^{n}{\left(y_{i}-\bar{y_{i}}\right)}^{2}}$$

(4) $$\%RMSEP=\frac{RMSEP}{range(y)}$$

, where *y_i_* is the *i^th^* measured value, ${\hat{y}}_{i}$ is the *i^th^* predicted value from the LOO cross-validation for model calibration or the *i^th^* predicted value for model validation, $\bar{y_{i}}$ is the mean of the measured values and *n* is the sample size in calibration or validation datasets. To account for model variation, jackknife permutation during model calibration was carried out. The derived coefficients were used to compute predicted values in validation datasets. From there, 95% confidence intervals of the predicted values were derived.

To test whether models trained on HSI data from one rice subpopulation or one nitrogen treatment level could be used to predict traits in the other subpopulation or treatment, follow-up PLSR models were developed for leaf-level *N* and *C*: *N*. Procedures for using the RReliefF algorithm and for building PLSR models were the same as described above (**Figure S5**). Additional analysis on leaf-level *N* was carried out to determine the effect of number of *W_PLSR_* and sample size on prediction metrics. This analysis aimed to sort out whether the number of wavelengths used as input to PLSR models described previously was appropriate. *W_PSLR_* at ten values (10, 50, 100, 150, 200, 300, 400, 500, 600 and 700) and sample sizes at five values (48, 60, 70, 78 and 88) were examined using one hundred iterations of each combination of these hyperparameters. We used a grid search method to evaluate this relationship (i.e., effects of sample size and wavelength number on prediction results); grid search was able to be leveraged as PLSR is more computationally tractable than advanced machine learning methods. *R* ^2^, *RMSEP* and %*RMSEP* were calculated and their means and standard errors were derived.

## 3 Results

### 3.1 Treatment and subpopulation effects on multivariate physiological traits

Examining pairwise relationships among the traits, we found that all traits had significant correlations with at least one other trait (*α* = 0.01), except for pre-dawn leaf-level net assimilation (*A*, *µmolm* ^−2^ *s* ^−1^) (**Figure S4**). As expected, traits that reflected similar aspects of physiology were more correlated. For example, nitrogen content (*N*, %) and leaf-level carbon to nitrogen ratio (*C*: *N*) had a strong negative correlation (-0.96), log transformed pre-dawn leaf-level transpiration (*log*(*E*)) and log transformed pre-dawn leaf-level stomatal conductance to water (*log*(*gsw*)) were perfectly positively correlated, and *pQY* (Fv/Fm) and *mQY* (Fv’/Fm’) had a weak positive correlation (0.48). We found that overall health status of the rice plants improved with nitrogen enrichment, as reflected by higher *pQY* and *mQY* in N1 (high nitrogen) than in N2 (low nitrogen) treatments. *N* was also found to have moderate and weak positive correlations with *pQY* and *mQY*, respectively (0.53 and 0.41). It is worth noting that specific leaf area with respect to dry weight (*SLA*, *cm* ^2^ *g* ^−1^ *dw*) had correlations with most of these traits; it had a weak negative correlation with *C*: *N*, *SLN*, and *log*(*E*) (-0.46, -0.34, and -0.27) and a weak positive correlation with *N*, *SLA_C_*, *mQY* and *pQY* (0.49, 0.42, 0.41, and 0.29). On the other hand, final biomass (*FB*, g) only had a weak negative correlation with *log*(*E*) and *log*(*gsw*) (-0.35 for both), suggesting that dry matter accumulation of rice in this experiment may have been more associated with water status than with nutrient status.

**Figure 2:**
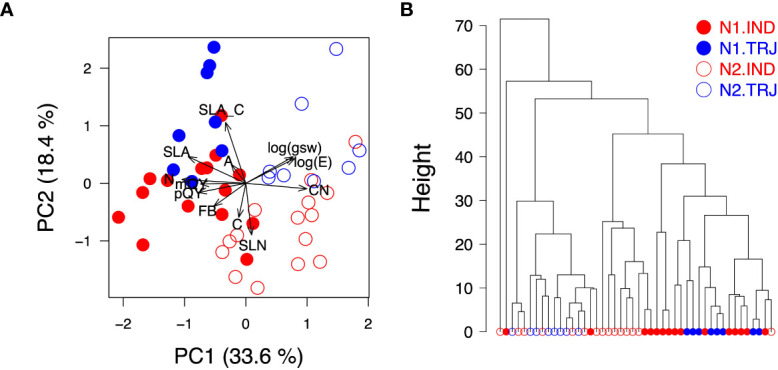
Treatment and subpopulation effects on physiological traits. **(A)** Principal components analysis biplot of PC1 (x-axis) and PC2 (y-axis). **(B)** Agglomerative hierarchical clustering using accessionmean physiological data. Open circles are observations from the low nitrogen treatment (N2); filled circles indicate the high nitrogen treatment (N1). Blue circles indicate accessions from the *tropical japonica* subpopulation (TRJ); red circles show accessions from the *indica* subpopulation (IND).

Due to the correlated nature of the ground-reference dataset, we employed two multivariate approaches to evaluate potential effects of subpopulation and treatment on high-level data structure. PCA on accessionmean trait data suggested both treatment and subpopulation effects (**Figure 2A**), with PC1 and PC2 reflecting treatment and subpopulation effects, respectively. The traits, *N* and *C*: *N*, contributed to PC1 (treatment) the most whereas *SLA_C_* and *SLN* had the most influence on PC2 (subpopulation). In contrast, hierarchical clustering showed a clear separation by treatment only and not subpopulation (**Figure 2B**). Interestingly, the dendrogram revealed that N2 *indica* observations were more closely clustered with the N1 group (both *indica* and *tropical japonica*) than with the cluster that primarily contained N2 *tropical japonica*, suggesting that *indica* may be less responsive to nitrogen enrichment than *tropical japonica*.

### 3.2 Effects of nitrogen on hyperspectral reflectance in rice

**Figure 3:**
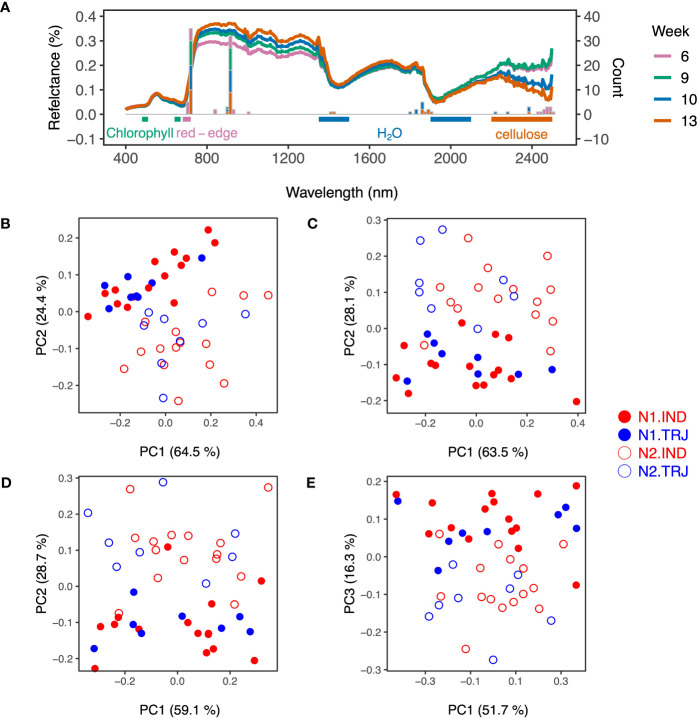
Variation in weekly hyperspectral imaging (HSI) reflectance data over plant developmental time. **(A)** Spectra show accession-mean HSI reflectance data (medians) across weeks. Stacked bar plots indicate key wavelengths identified based on the top 20 - 30 loadings in principal component analysis (PCA) from each week. Horizontal bars represent regions that are sensitive to chlorophyll concentration (green; 480 – 510 nm and 640 – 670 nm), water content (blue; 1350 – 1500 nm and 1900 - 2100 nm) and cellulose (orange; 2200 – 2500 nm). The pink horizontal bar marks the Red Edge region (680 - 720 nm). Plots shown in **(B–E)** are results from PCA conducted on accession-mean HSI reflectance data on Weeks 6, 9, 10 and 13, respectively.

Weekly accession mean HSI data gave rise to spectra typical of plants (**Figure 3A**). Due to the high dimensionality and correlated nature of these data, PCA was applied on accession-mean HSI datasets to examine the potential effects of nitrogen treatment and subpopulation identity. Similar to results of hierarchichal clustering of the ground-reference data, only the treatment effect was clear throughout the time period analyzed (**Figures 3B–E**); separation of treatment levels was primarily determined by PC2 from Weeks 6 to 10 and by PC3 on Week 13. However, nitrogen treatment signals appeared to be lower on Week 13 than on earlier weeks.

Using these PCA results, wavelengths were selected for use in support vector machines (SVM); these are referred to hereafter as *W_SVM_* (see Materials and Methods for details). *W_SVM_* detected in PC2 from Weeks 6 to 10 were similar to those detected in PC3 on Week 13 and were all centered around 715 nm (**Figure 3A**) in the Red Edge region, indicating that the treatment signals could be attributed to similar spectral regions across the full experimental period. A detailed examination showed that wavelengths around 715 and 910 nm accounted for the highest and the second highest proportions of *W_SVM_*, respectively. Wavelengths around 2200 – 2400 nm, a highly variable region, was found to contribute to the set of *W_SVM_* as well. This was observed for all weeks except Week 9. Lastly, *W_SVM_* of Weeks 10 and 13 included wavelengths around 1400 and 1800 nm, a region known to be informative of water absorption.

**Figure 4:**
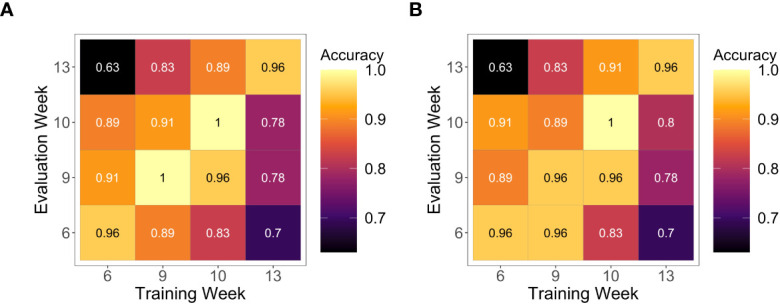
Nitrogen treatment levels predicted by support vector machines. Classification results from support vector machines using **(A)** a radial basis function kernel and **(B)** a linear kernel. These were trained using *W_SV M_* from the training week.

We next quantified treatment classification accuracy of these selected wavelengths by applying SVM. Results showed that *W_SVM_* were able to classify nitrogen treatments for any one week using information from any other weeks (**Figure 4**). Overall, accuracies ranged from 0.63 to 0.91, with accuracy greatest for Weeks 6 through 10 and poorest for Week 13. Accuracy of prediction made by Week 13 dropped as evaluation weeks became more temporally distant from the training week; the lowest accuracy using the Week 13 classifier was observed for Week 6 (0.7 for both kernels) while for Weeks 9 and 10, the accuracy was about 0.78.

### 3.3 Use of hyperspectral imaging as surrogates for physiological traits in rice

Based on promising results of the SVM classification, we next wanted to test the utility of HSI-derived data to estimate trait values. The RReliefF algorithm was first used to derive *W_PLSR_*, *i.e.* wavelengths selected for partial least squares regression (PLSR) (Materials and Methods). Reflectance data of *W_PLSR_* were then used directly as the predictors of single-response PLSR models. Model results are summarized below based on several trait categories: biomass constituent, growth, photosynthetic capacity, and water transport.

#### 3.3.1 Biomass constituent traits

**Figure 5:**
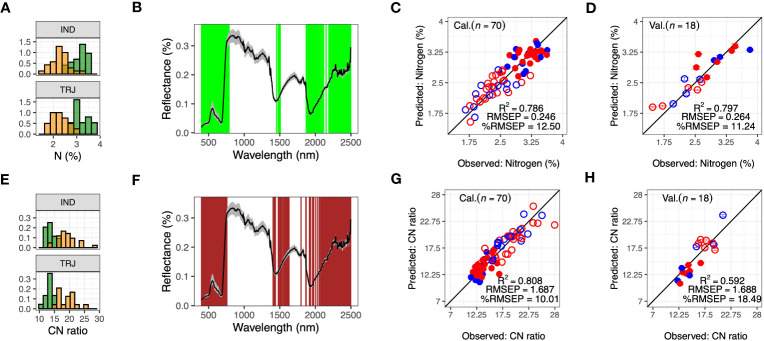
Predicting leaf-level nitrogen (*N*) and CN ratio (*C*: *N*) using hyperspectral reflectance imaging data. **(A)** Distribution of leaf-level nitrogen (*N*) in *indica* (IND) and *tropical japonica* (TRJ) accessions under high nitrogen (N1, green) and low nitrogen (N2, yellow) treatments, **(B)** Selected wavelengths (green lines) for predicting *N* from Week 9 HSI data. **(C, D)** are model calibration and validation for predicting *N*, respectively. **(E)** Distribution of *C*: *N* in IND and TRJ in N1 (green) and N2 (orange) treatments, **(F)** the selected wavelengths (dark red lines) for predicting *C*: *N* from the weekly averaged HSI data, and **(G, H)** are model calibration and validation for predicting *C*: *N*, respectively. Error bars are 95% confidence intervals. In **(B)** and **(F)**, black lines show reflectance values and gray shaded areas indicate reflectances between the 5*^th^* and the 95*^th^* percentiles. In panels **(C, D, G, H)**: Open circles = N2 treatment; filled circles are N1 treatment; red = IND and blue = TRJ.

Ground-reference observations for biomass constituent traits (leaf-level carbon (*C*, %), nitrogen (*N*, %) and C:N ratio (*C*: *N*)) were collected during Week 9. Distributions of *N* and *C*: *N* were aligned with nitrogen treatment (**Figures 5A, E**), and *W_PLSR_* for both *N* and *C*: *N* were composed of 400 wavelengths; these sets were similar to each other and included wavelengths from the VIS and Red Edge regions and covered wavelengths around 1500 nm and 2000 nm, regions sensitive to water inside plant cells (**Figures 5B, F**). In calibration models, *R* ^2^ for *C*: *N* and *N* were 0.808 and 0.786, respectively (**Figures 5C, G**, **S6**). *RMSEP* were 1.687 and 0.246 (%), respectively, and %*RMSEP* were 10.01% and 12.50%, respectively. For model validation, validation of *N* models (*R* ^2 = ^0.797, *RMSEP* = 0.264 and %*RMSEP* = 11.24) were better than validation of *C*: *N* models (*R* ^2 = ^0.592, *RMSEP* = 1.688 and %*RMSEP* = 18.49) (**Figures 5D, H**). In contrast, PLSR models could not be built with *C* as the lowest RMSEP during LOO-calibration suggested that a 0-component model was the most appropriate.

#### 3.3.2 Growth traits

Traits of this group included specific leaf area with respect to leaf dry weight (*SLA*, *cm* ^2^ *g* ^−1^), specific leaf area with respect to carbon (*SLA_C_*, *cm* ^2^ *mg* ^−1^(*C*)), specific leaf nitrogen (*SLN*, *mg*(*N*)*cm* ^−2^), final biomass (*FB*), and pre-dawn leaf-level net assimilation (*A*, *µmolm* ^−2^ *s* ^−1^), which reflects dark respiration. *FB* was normalized and *SLN* was log transformed, denoted as *norm_FB_*, and *log*(*SLN*), respectively, prior to building prediction models. PLSR modeling results are summarized in **Figure S7**. *W_PLSR_* across the three traits were similar in that they did not include wavelengths in the green light region (520 – 600 nm) but did include the Red Edge region. Compared to *W_PLSR_* for *N* and *C*: *N*, *SLA*, *SLA_C_* and *log*(*SLN*) included *W_PLSR_* in NIR plateau region. We found that calibration models for these three traits were not ideal (*R* ^2^ ≤ 0.295, %*RMSEP* ≥ 14.00) and that their validation models were unstable as model metrics varied greatly when datasets were permuted to generate different combinations of calibration and validation datasets. For *FB* and *A*, both their calibration and validation models were very poor (**Figure S7**).

#### 3.3.3 Photosynthetic capacity and water transport traits

Photosynthetic capacity traits included pre-dawn quantum yield Fv/Fm (*pQY)*, midday quantum yield Fv’/Fm’ (*mQY)* and *V_cmax_* and *J_max_*. *pQY* and *mQY* were normalized prior to building models, denoted as *norm_m_QY* and *norm_p_QY*, respectively. Calibration models were not very predictive with *R* ^2^ ≤ 0.344, *RMSEP* ≥ 0.832, and %*RMSEP* ≥ 14.91, and validation models for both traits revealed that the models were not applicable (*R* ^2^ ≤ 0.19 and %*RMSEP* ≥ 20.90) (**Figure S8**). *W_PLSR_* for *V_cmax_* and *J_max_* spanned nearly the entire spectral region examined, and similar to the performance of growth trait models, these models were not stable as when the datasets were permutated or could not be established. Likewise, PLSR models could not be established for water transport traits, *log*(*E*) and *log*(*gsw*) (**Figure S9**).

#### 3.3.4 Trait predictions in one treatment level using models developed from another

**Figure 6:**
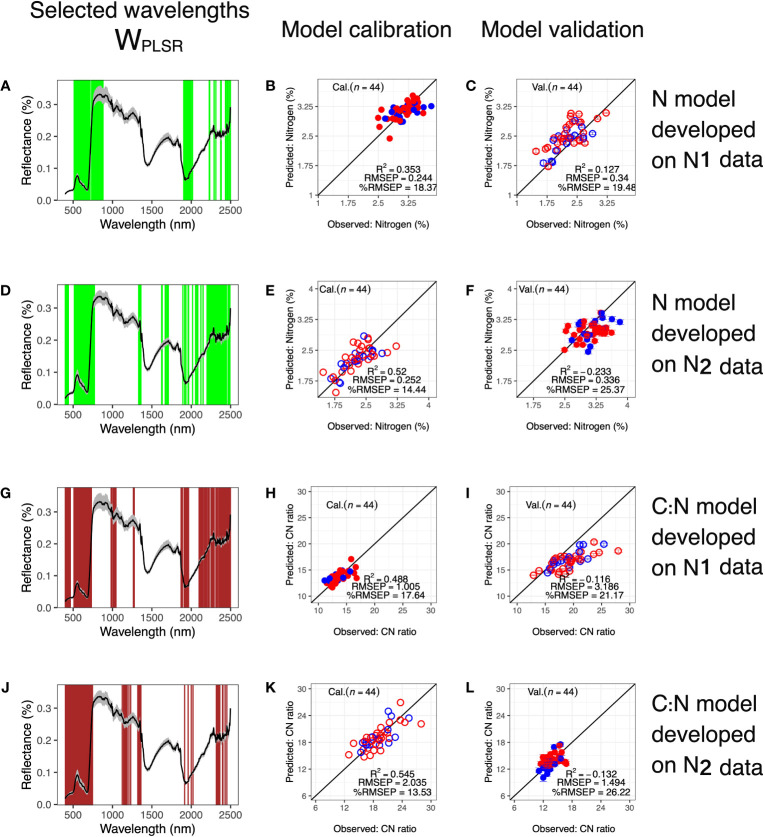
Predicting traits in one treatment level using models developed from another. Wavelengths selected based on calibration datasets of **(A)** *N* data in N1 treatment, **(D)** *N* data in N2 treatment, **(G)** *C*: *N* data in N1 treatment and **(J)** *C*: *N* data in N2 treatment, respectively. Corresponding calibration results are in **(B, E, H, K)**, respectively. Calibrated models were used to predict **(C)** *N* in N2 treatment, **(F)** *N* in N1 treatment, **(I)** *C*: *N* in N1 treatment and **(L)** *C*: *N* in N2 treatment, respectively. In **(A, D, G, J)**, black lines show mean reflectance values and gray shaded areas indicate reflectances between the 5^th^ and the 95^th^ percentiles. In calibration and validation plots: Open circles = N2 treatment; filled circles = N1 treatment; red = IND and blue = TRJ.

As models to predict leaf-level *N* and *C*: *N* from HSI data performed well, we further examined whether we could develop models using only one treatment level to predict traits in the other treatment level, termed treatment-based models. Due to the smaller sample size for model calibration, sizes of *W_PLSR_* shrunk to 300. *W_PLSR_* for the treatment-based models generally covered the same wavelengths as *W_PLSR_* for model calibration utilizing the full dataset. However, treatment-based models tended to have *W_PLSR_* more dispersed in NIR and SWIR regions, especially for models built from the N2 treatment level (**Figure 6**). In addition, while *W_PLSR_* from full datasets of *N* and *C*: *N* included wavelengths around 1350-1500 nm, treatment-based models omitted wavelengths in this region, possibly due to the smaller number of wavelengths considered (300 vs 400). Calibration for treatment-based models performed moderately well with *R* ^2^ ≥ 0.35, and %*RMSEP* ≤ 18.37. However, it was apparent from validation results that models developed using data from one treatment should not be applied to predict samples in the other treatment (*R* ^2^ ≤ 0.127 and %*RMSEP* ≥ 19.48).

#### 3.3.5 Trait predictions in one subpopulation using models developed from another

Rice has two deeply diverged subpopulations, both of which were represented in this study, so we next examined whether models developed from one subpopulation could be used to predict leaf-level *N* and *C*: *N* in the other subpopulation. These are termed subpopulation-based models. In the case of *N*, *W_PLSR_* for subpopulation-based models appeared similar and included wavelengths in the VIS and Red

**Figure 7:**
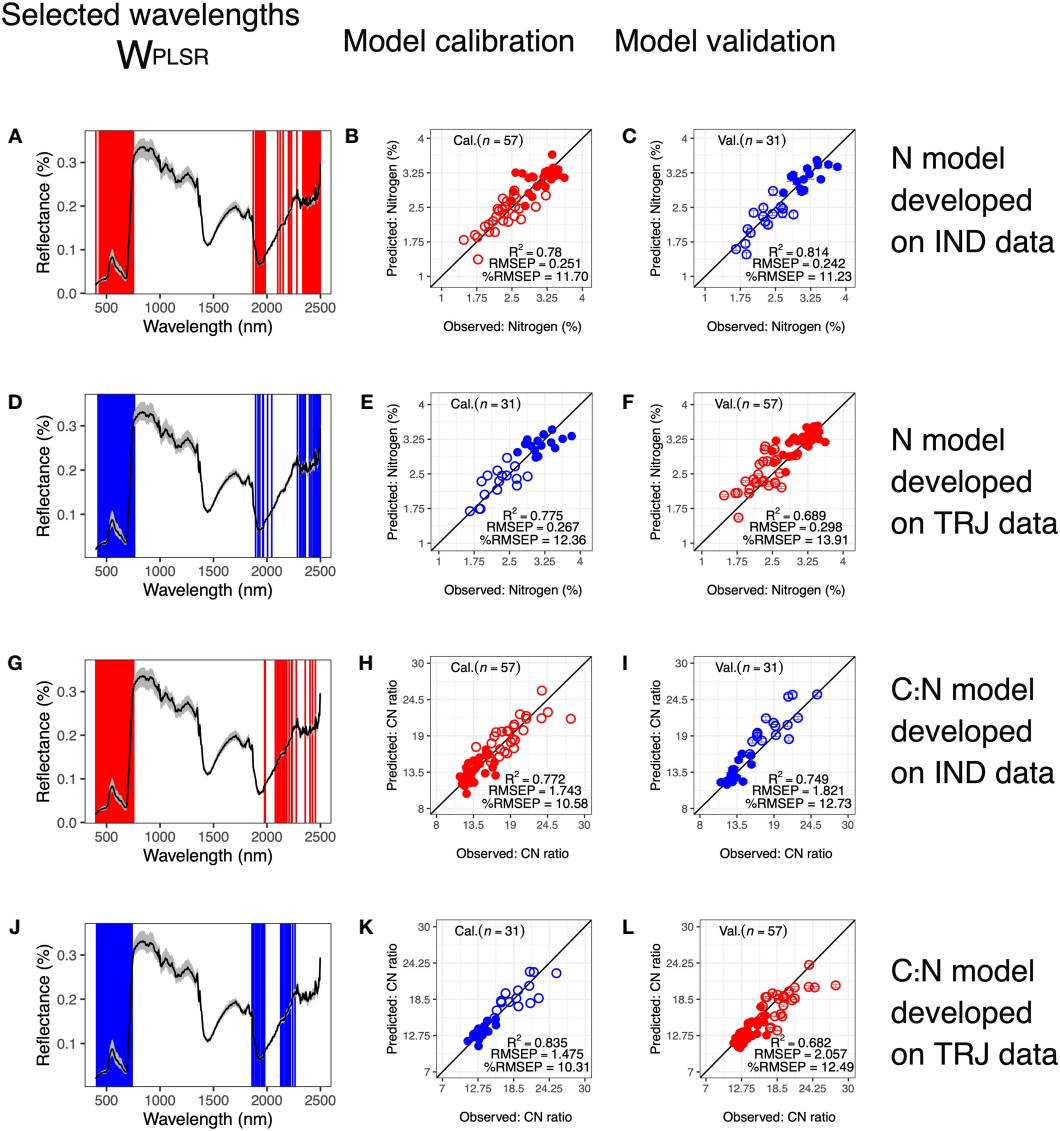
Predicting nitrogen (*N*) and CN ratio (*C*: *N*) in one subpopulation using models developed from another. Wavelengths selected based on calibration datasets of **(A)** *N* data in IND subpopulation, **(B)** *N* data in TRJ subpopulation, **(G)** *C*: *N*data in IND subpopulation and **(J)** *C*: *N* data in TRJ subpopulation, respectively. Corresponding calibration results are in **(B, E, H, K)**, respectively. Calibrated models were used to predict **(C)** *N* in TRJ subpopulation, **(F)** *N* in IND subpopulation, **(I)** *C*: *N* in TRJ subpopulation and **(L)** *C*: *N* in IND subpopulation, respectively. In **(A, D, G, J)**, black lines show mean reflectance values and gray shaded areas indicate reflectances between the 5*^th^* and the 95*^th^* percentiles. In calibration and validation plots: Open circles = N2 treatment; filled circles = N1 treatment; red = IND and blue = TRJ.

Edge regions, wavelengths around 1900 – 2100 nm, and wavelengths at the end of the SWIR region (**Figures 7**, **S10**). Both model calibration and validation models performed well, with performance similar to that of models developed from the full dataset (calibration: *R* ^2^ ≥ 0.775, *RMSEP* ≤ 0.671 and %*RMSEP* ≤ 12.36; validation: *R* ^2^ ≥ 0.689, *RMSEP* ≤ 0.298 and %*RMSEP* ≤ 13.91).

For the subpopulation-based *C*: *N* models, *W_PLSR_* was similar to those of the subpopulation-based *N* models (**Figure 7**), with the exception of when TRJ was used for model calibration, *W_PLSR_* did not include any wavelengths greater than 2300 nm. This differentiated it from the other three subpopulation-based models. All subpopulation-based models excluded wavelengths around 1350-1500 nm, which were used in full dataset models (**Figure 5**). Model calibration and validation performed well in both subpopulationbased models for *C*: *N*(calibration: *R* ^2^ ≥ 0.772, *RMSEP* ≤ 1.743 and %*RMSEP* ≤ 10.58; validation: *R* ^2^ ≥ 0.682, *RMSEP* ≤ 2.057 and %*RMSEP* ≤ 12.49). Comparing models developed from the full dataset with subpopulation-based models, the three model metrics (*RMSEP*, *R* ^2^, and %*RMSEP*) did not point to the same conclusion, *i.e.* high *R* ^2^ in one model did not correspond with low *RMSEP* or low %*RMSEP*. From the perspective of *RMSEP*, models developed with data from IND had better calibration results compared with models developed from the full dataset or with model developed from data in TRJ subpopulation. However, validation results from the full dataset remained the best, as may be expected given its greater variability around the mean. *RMSEP* from validation for full dataset was 0.133 and 0.369 lower than *RMSEP* from models developed from IND and TRJ data, respectively.

#### 3.3.6 Effect of sample size and number of wavelengths on model performance

**Figure 8:**
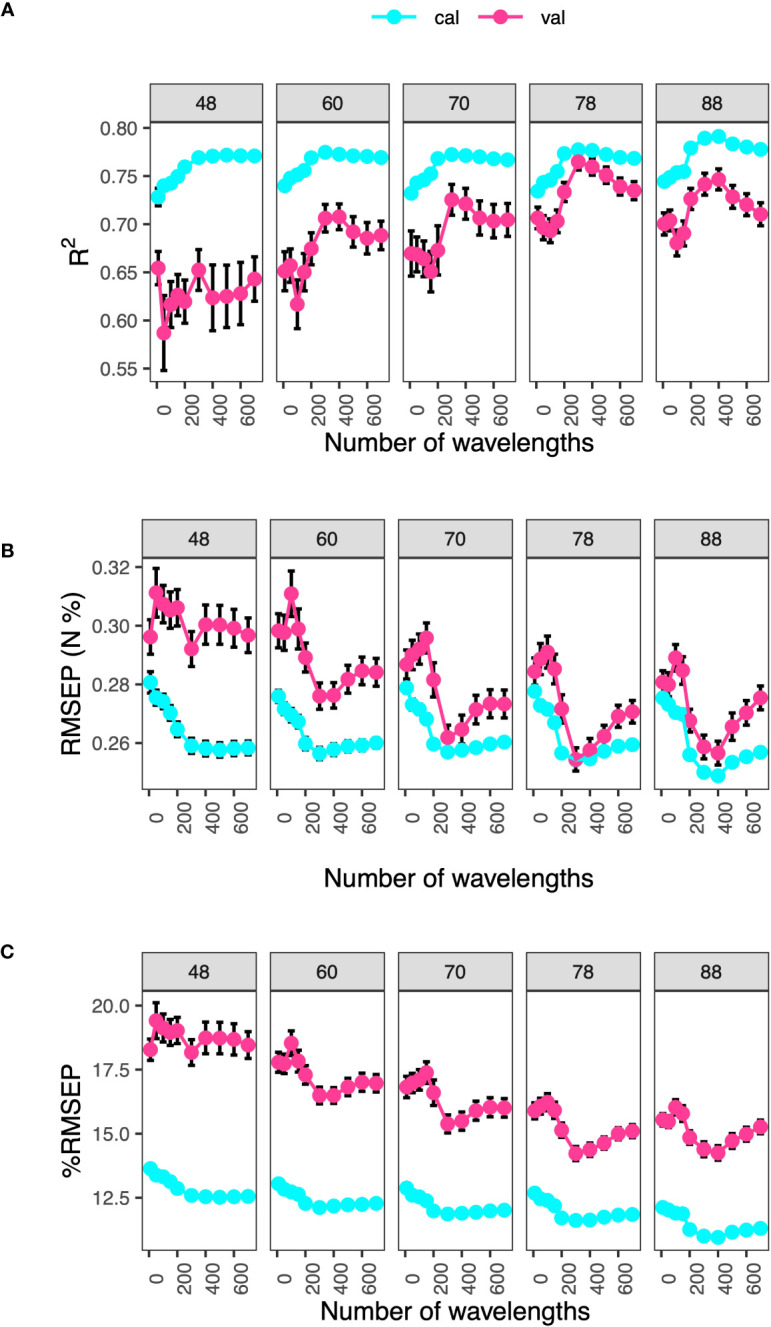
Effect of sample size and number of wavelengths on model performance. Model metrics, **(A)** *R* ^2^, **(B)** RMSEP (N, %) and **(C)** %*RMSEP*, are derived from nitrogen prediction models. Selected wavelengths are those with the top rankings based on the RReliefF algorithm in calibration datasets. Labels denote total sample sizes, with 80% of it used for model calibration. Error bars are standard errors from 100 iterations.

We next investigated the effects of sample size and number of wavelengths on model performance using *N* as the target trait (**Figure 8**). Results showed that when total sample size was greater than 70, models with 300 or 400 wavelengths had the best performance. When the number of wavelengths were greater than 400, model performance dropped as indicated by the decrease in *R* ^2^ (increase in *RMSEP* and %*RMSEP*) in model validation. This suggested that: (1) We needed at least total sample size of 78, which was equivalent to 62 samples in model calibration, to afford model complexity, and (2) models suffered from over-fitting when number of wavelengths were greater than 400. In addition, we noticed that when the total sample size was less than 60, standard errors of the model metrics in validation tended to be large; this meant models were too complex. Lastly, from the perspectives of *R* ^2^ and *RMSEP*, sample sizes of 48 and 60 caused under-fitting, observed as large differences in model metrics between validation and calibration. Model performance started to converge when total sample sizes were greater than 78. On the other hand, %*RMSEP* did not converge as much as for *R* ^2^ or *RMSEP*. This may be due to the fact that %*RMSEP* was a normalized metric and is more robust than *R* ^2^ or *RMSEP*.

## 4 Discussion

In this study, we leveraged the genetic divergence of two rice subpopulations along with two levels of nitrogen application to drive the range of phenotypic variation observed for a suite of physiological traits and hyperspectral images. As previous work relating spectral features to rice traits focused on limited sets of varieties, our overarching goal was to assess the potential application of hyperspectral reflectance as trait surrogates across diverse rice accessions. The relatively long experimental period (13 weeks) additionally afforded an opportunity to ask about the persistence of HSI utility over the course of plant development.

Overall, we found that the traits, *N* (leaf-level nitrogen content) and *C*: *N* (leaf-level carbon to nitrogen ratio) could be predicted using HSI data, whereas for other traits, models either could not be developed or resulted in poor validation. Of note, *N* and *C*: *N* were traits that contributed most to the first principal component of the PCA using ground-reference data, which separated nitrogen treatment classes, and we speculate that this relationship contributed to the high performance of the PLSR models for these traits. Previous work has also demonstrated successful *N* prediction in crop species using HSI data (Vigneau et al., 2011; Homolová et al., 2013; Din et al., 2017; Tan et al., 2018; Bruning et al., 2019; He et al., 2020; Meacham-Hensold et al., 2020; Vergara-Diaz et al., 2020; Yu et al., 2020; Baath et al., 2021; Lin et al., 2022). Under various growth conditions, reflectance variation in the VIS and Red-Edge regions along with the end of the SWIR region have been identified as important for *N* prediction; our findings were consistent with these previous studies (**Figure 5**). The close relationship between *N* and chlorophyll may explain why model prediction is often successful, as plant spectral features in the VIS region are largely determined by pigments. In contrast with *N*, *C*: *N* has not been frequently reported as a predictable trait from hyperspectral reflectance. Gao et al. (2020) found that predicting *C*: *N* was related to wavelengths around the red and Red-Edge regions (560 nm, 600 nm, 660 nm, 680 nm, 720 nm, 760 nm and 1470 nm) of forage-grass in the Tibetan Plateau. The *W_PLSR_* for the full *C*: *N* model as well as subpopulation-based models largely overlapped with the wavelengths that previous study had identified, except for 760 nm, which was not in *W_PLSR_* for TRJ model, and 1470 nm, which was not found in either TRJ- or IND-based models. Our results showed that *W_PLSR_* between *N* and *C:N* were similar (**Figure 5**, consistent with the high correlation between the two traits **Figure S4**). This observation suggests that interpretation of selected wavelengths should be made with caution, as they may not represent cause-effect relationships with the target trait.

HSI data were unable to predict the other traits evaluated in this study. In the case of leaf-level carbon, *C*, past literature also concluded that *C* was not predictable Gao et al. (2020), even though specific chemical compounds like lignin, cellulose and starch could be inferred from spectral data (Curran, 1989). We speculate that while those chemical compounds are responsive to specific wavelengths, signals disappear when all of the compounds are considered at once, as in the case of *C*. For *FB*, the inability to predict this trait using HSI data sits in contrast to previous research that was able to leverage HSI to predict biomass (Cho et al., 2007). Since *FB* was only collected at the end of the experimental period in our study, there may have been too much architectural complexity by that late stage in development. In this case, image transformation may be helpful to amelerioate the influence of plant architecture on spectral characteristics, and previous studies have reported various methods to extract relevant information from complex HSI data (Al Makdessi et al., 2019; Asaari et al., 2019; Mishra et al., 2020; Mertens et al., 2021). Additionally, the observed variation of *FB* between the two treatments in our study was small (**Figure S7M**). Collecting biomass data at multiple time points would increase the overall size and variation of the ground-reference dataset, which would likely improve model performance. However, this requires more destructive sampling, which was not amenable to the experimental design considered here.

For other traits, we surmise that one general underlying reason for the inability to build satisfactory models is due to the differences in spatial scale between imaging data (taken at canopy-level) and ground-reference observations (taken at leaf-level of the youngest fully expanded leaf of a single tiller). For these particular traits, heterogeneity among individual leaves is likely present, which would not have been captured by our ground-reference observations. Previous research successfully predicted *SLA* from HSI data by utilizing a hand-held spectroradiometer on the same individual leaves used for ground-reference measurements (Cotrozzi et al., 2020). To improve model performance, future work using canopy-level HSI systems should strive to capture some intra-canopy variation in ground-reference trait data. Similarly, temporal lags between ground-reference measurements and imaging events should be minimized, especially for traits that represent fast processes in plants that are very sensitive to environmental differences Ge et al. (2016). In our particular setup at the AAPF, plants travel on conveyor belts out of the growth chamber environment to the imaging booth (**Figure S2**). This takes approximately four minutes, during which temperature drops to around 21 °C. A sensors-to-plants approach would alleviate the effects of temporal decoupling Yang et al. (2020), however, this would depend on available infrastructure. Lastly, Meacham-Hensold et al. (2019) and Fu et al. (2020) were able to predict *V_cmax_* and *J_max_* in tobacco with PLSR. Compared to the current work, these past studies had many more observations; we speculate that our dataset did not have enough variation in A/Ci curves for successful PLSR predictions.

One question we were keen to address here was whether HSI signatures differed in accordance with subpopulation identity. We determined that, despite clear subpopulation differentiation in ground-reference trait data, there were no background genetic signatures in HSI information for rice, based on PCA and model predictions of *N* and *C*: *N* (**Figures 3**, **7**). This has several implications for moving forward in conducting larger-scale experiments leveraging HSI-predicted physiological traits in diverse rice: (1) subpopulation genetic background does not need to be controlled for in prediction models; and (2) calibration models can be developed from a combination of genetic materials, similar to NIR models for stem non-structural carbohydrate traits in rice (Wang et al., 2016b), conditional that there is adequate phenotypic variation. Contrary to our results, He et al. (2020) suggested that *indica* and *japonica* subpopulations required different models to estimate *N*. The main design difference is that their study utilized top-view HSI data, and they analyzed only derived Vegetation Indices. In contrast, we utilized side-view data and analyzed spectral data directly. One important point to make is that our experiment was carried out only during the vegetative stage of rice. Therefore, we speculate that these results may not extend to rice during the reproductive stage; there are several reasons: (1) occlusion of rice leaves or panicles would likely be more apparent in reproductive stage than in early vegetative stage (Din et al., 2017). In addition, some genotypes exert more panicles than the others (Wang et al., 2016a). And (2), during the reproductive stage, resources in leaves are transferred to grains, which would likely lead to changes in hyperspectral signature of leaves.

Automated phenotyping systems, such as Purdue’s AAPF, readily support high frequency of hyperspectral imaging, as imaging events can be programmed prior to the start of the experiment and require limited human intervention (**Figure S2**). In our study, these events occurred two to three times per week throughout the experimental period. The frequency of imaging allowed us to average across several days to obtain pot-level spectral information to be used for downstream analysis and modeling; this attenuates potentially noisy individual spectra and helps detect the overall HSI signature of each plant during timepoints of interest. Having automated imaging occur from Week 6 through Week 13 also enabled us to ask about the consistency of HSI information over developmental time. To test this, we employed SVM to see if HSI could classify nitrogen treatments forwards and backwards in time (*i.e.*, using one week to predict another week). Classification using HSI data performed very well overall (**Figure 4**), and prediction accuracies were greater than 0.83, excluding Week 13. Accuracies where Training and Evaluation Weeks were the same (diagonals in **Figure 4**), however, may be over-estimated due to overlap in datasets. Additional improvements in classification accuracy of nitrogen treatment could consider (1) testing different feature selection methods prior to SVM; (2) analyzing hyperspectral images directly, such as using Fourier Transformation to extract spectral features; or (3) using different classification methods such as CNN (Singh et al., 2016).

It is interesting to note that the latest timepoint, Week 13, showed the poorest performance out of all weeks, no matter whether it served as the Training or the Evaluation Week. This timepoint occurred at the very end of the experimental period, and we speculate that water may have played an interactive role with the nitrogen treatment to modify HSI signatures. We found that higher levels of nitrogen led to greater *SLA* in our experiment (**Table S3**), meaning greater transpiring leaf area per unit dry biomass; this translates to an increased demand. Watering regime, which was managed on a target weight basis using the facility’s automated system, did not differ between the two nitrogen treatments; therefore, it is plausible that the high nitrogen treatment would have experienced some level of water stress by the end of the experiment. This explanation is supported by the observation that on Week 13, key wavelengths derived from PCA showed a shift from 2400 nm to 1800 nm, adjacent to the region known to be responsive to water (**Figure 3A**), and we noticed leaf-rolling (O’Toole and Cruz, 1980) in some plants during midday in the latter part of the experimental period.

With the establishment of automated plant phenotyping systems, the promise of hyperspectral images to serve as surrogates for plant physiological traits has motivated a wide range of studies across different species and treatments over the last decade. However, it is important to recognize that these image-derived reflectance data are influenced by multiple factors such as plant architecture, scaling, equipment (e.g., cameras), and the particular setup of each facility, which impacts the timing of imaging as plants move from their growing environment to imaging booths. For these reasons, prediction models are generally considered non-transferrable across species, experiments, or facilities (Cho et al., 2007; Verrelst et al., 2015). Flexible approaches to designing within-experiment calibration sets with robust ground-reference data are therefore needed; general frameworks such as RReliefF-PLSR prediction coupled with the optimization of hyperparameters for model development, as presented here, can be extended beyond any single study. As image processing methods continue to advance, researchers may be able to more readily extract reflectances of individual leaves from canopy-level images; this would enable plant scientists to explore within-plant trait distributions and how that varies across genotypes. These kinds of insights can help deepen understanding of plant physiological response mechanisms across intra-specific genetic variation.

## Data availability statement

The datasets presented in this study can be found in online repositories. The names of the repository/repositories and accession number(s) can be found below: https://purr.purdue.edu/publications/4079/.

## Author contributions

AS, CG, CH, YY, and DW conceptualized the study. RI, CH, and DW collected data. AS and T-CT analyzed data. T-CT and DW wrote the manuscript and all authors contributed feedback and/or edits. All authors contributed to the article and approved the submitted version.
