# Supplementary material for: Quantifying physiological trait variation with automated hyperspectral imaging in rice
Source: Front Plant Sci. 2023 Sep 20;14:1229161. doi: 10.3389/fpls.2023.1229161 (PMC10548215; doi:10.3389/fpls.2023.1229161)
Supplement: Supplementary file 1 [file DataSheet_1.pdf]

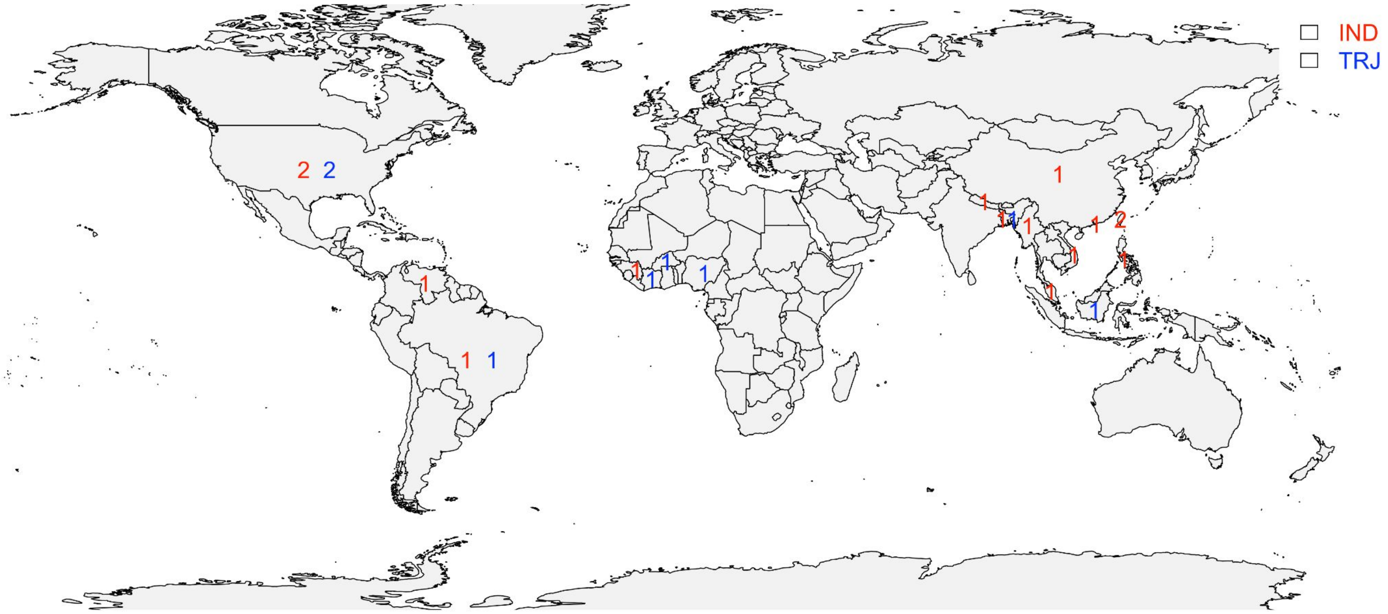

Figure S1. Geographical distribution of 23 rice accessions evaluated this study. Number on the map indicates the number selected accessions from each subpopulation in each country of origin. Color indicates subpopulation identity: red = *indica* (IND) and blue = *tropical japonica* (TRJ).

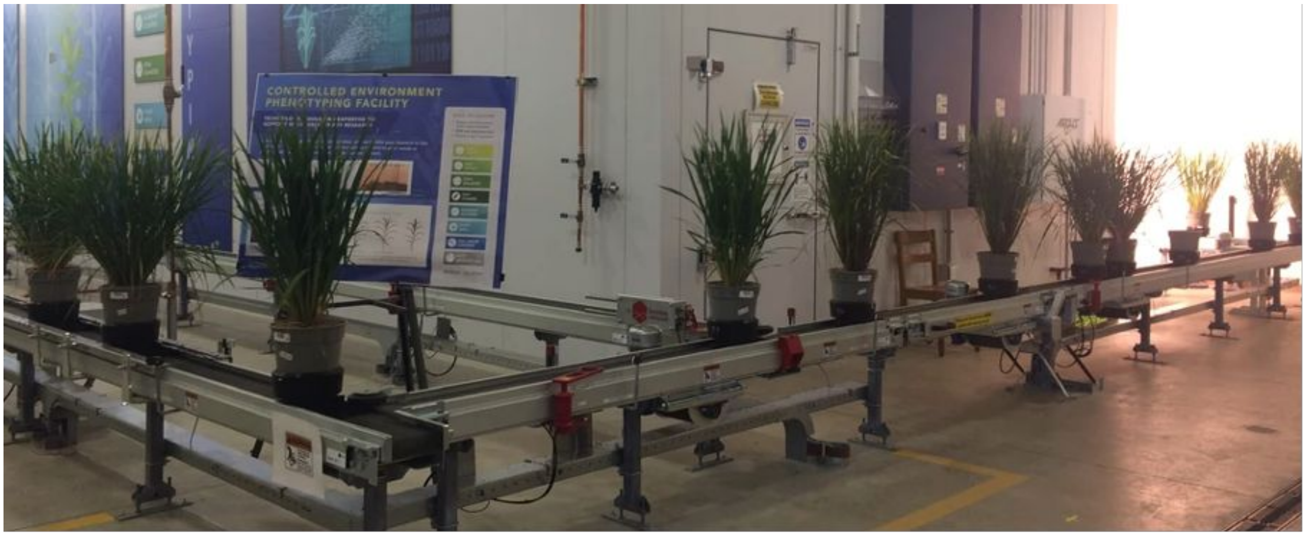

Figure S2. Ag Alumni Seed Phenotyping Facility. Rice plants traveling on conveyor belt outside of the growth chamber. Bright light in the upper-right hand side of photo shows where the door to the growth chamber is located.

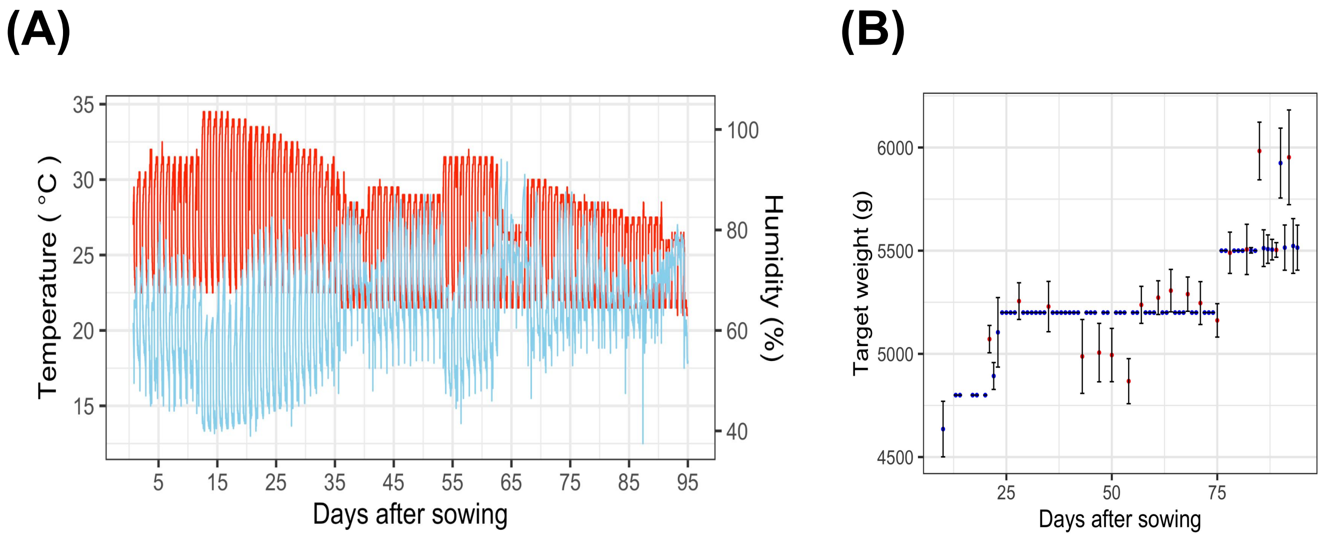

Figure S3. Growth condition history over the course of the experiment in the Ag Alumni Seed Phenotyping Facility. (A) 10-minute interval of temperature (red) and humidity (blue). Data presented are the averages of seven loggers placed on pots randomly inside the facility. (B) Irrigation history. Blue and red points indicate when auto-irrigation and fertigation occurred, respectively (errors bars are standard deviation of 94 plants). Irrigation was based on target weight, which was the same among all pots (*i.e.*, individual plants) whereas fertigation was based on nitrogen concentration (volume). The fertigation schedule is as follows: 200 ml on 21 days after sowing (DAS); 400 ml on 28 DAS; 600 ml on 35 DAS; 600 ml twice *per* week from 43 to 54 DAS; 900 ml twice *per* week from 57 to 75 DAS; 1200 ml twice *per* week from 78 to 94 DAS. Since target weights were never set above pot water holding capacity, the chance of leaching was minimized.

(A)

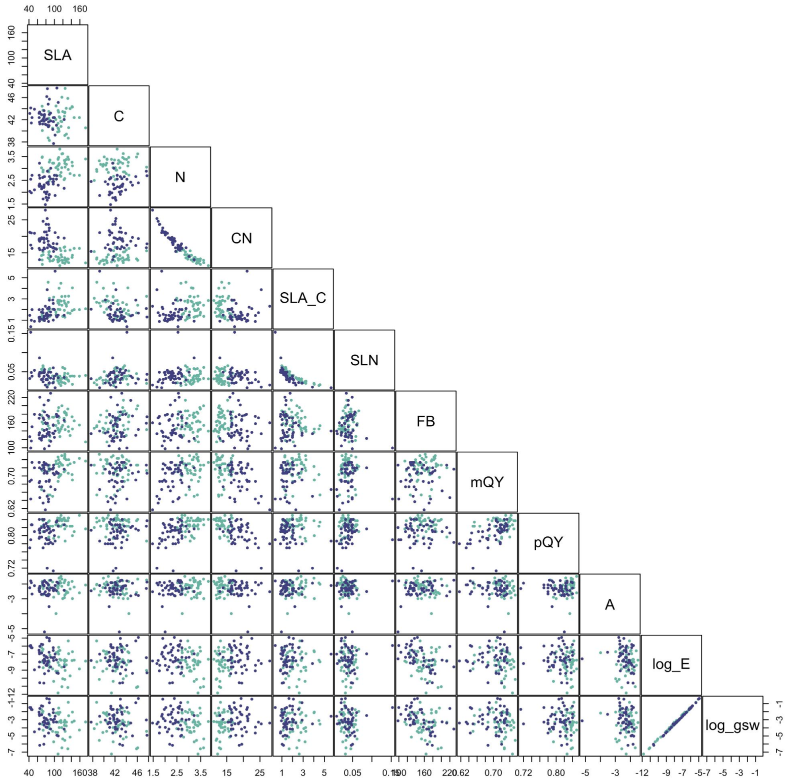

(B)

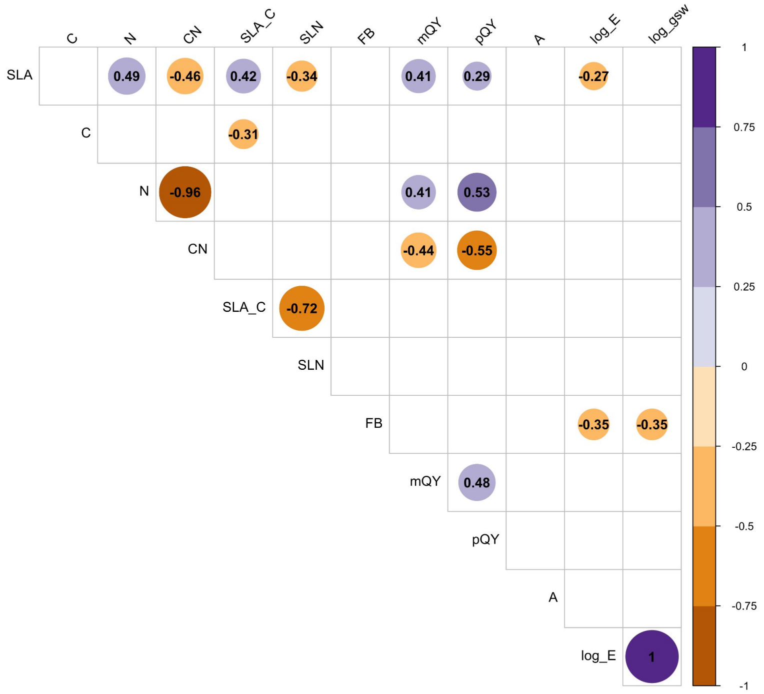

Figure S4. Pairwise relationship among the ground-reference physiological traits. (A) Pairwise scatterplots. High nitrogen (N1) and low nitrogen (N2) treatments are colored with green and purple, respectively, and (B) pairwise pearson correlation coefficients. Only the significant correlations are shown (alpha = 0.01).

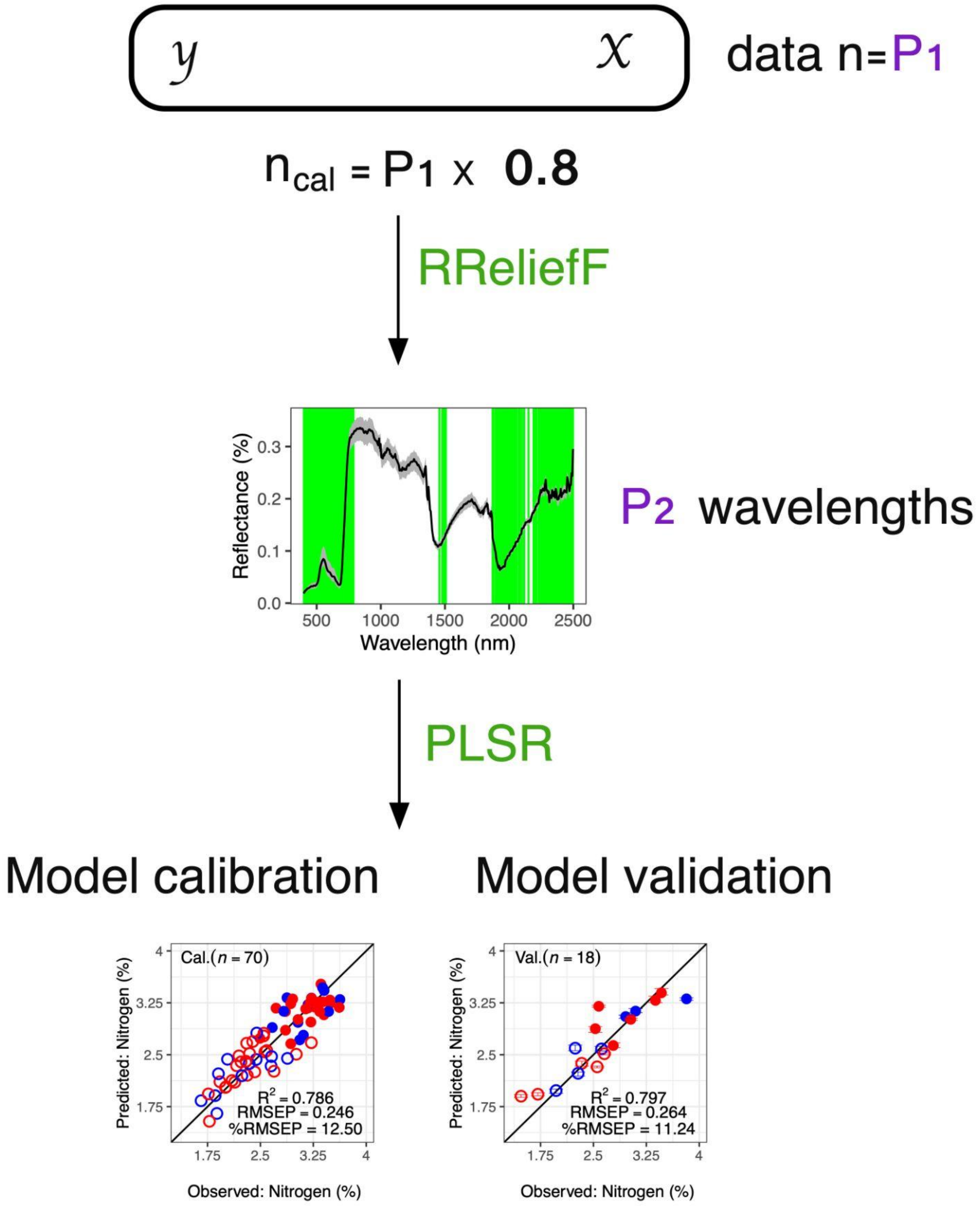

Figure S5. Pipeline for developing PLSR models. To predict a rice physiological trait (y) from high dimensional and correlated hyperspectral data (X), RReliefF is first applied to select wavelengths as predictors W_{PLSR} in partial least squares regression (PLSR) model. Model performance is influenced by total sample size P1 and selected number of wavelengths from the evaluation of reliefF P2.

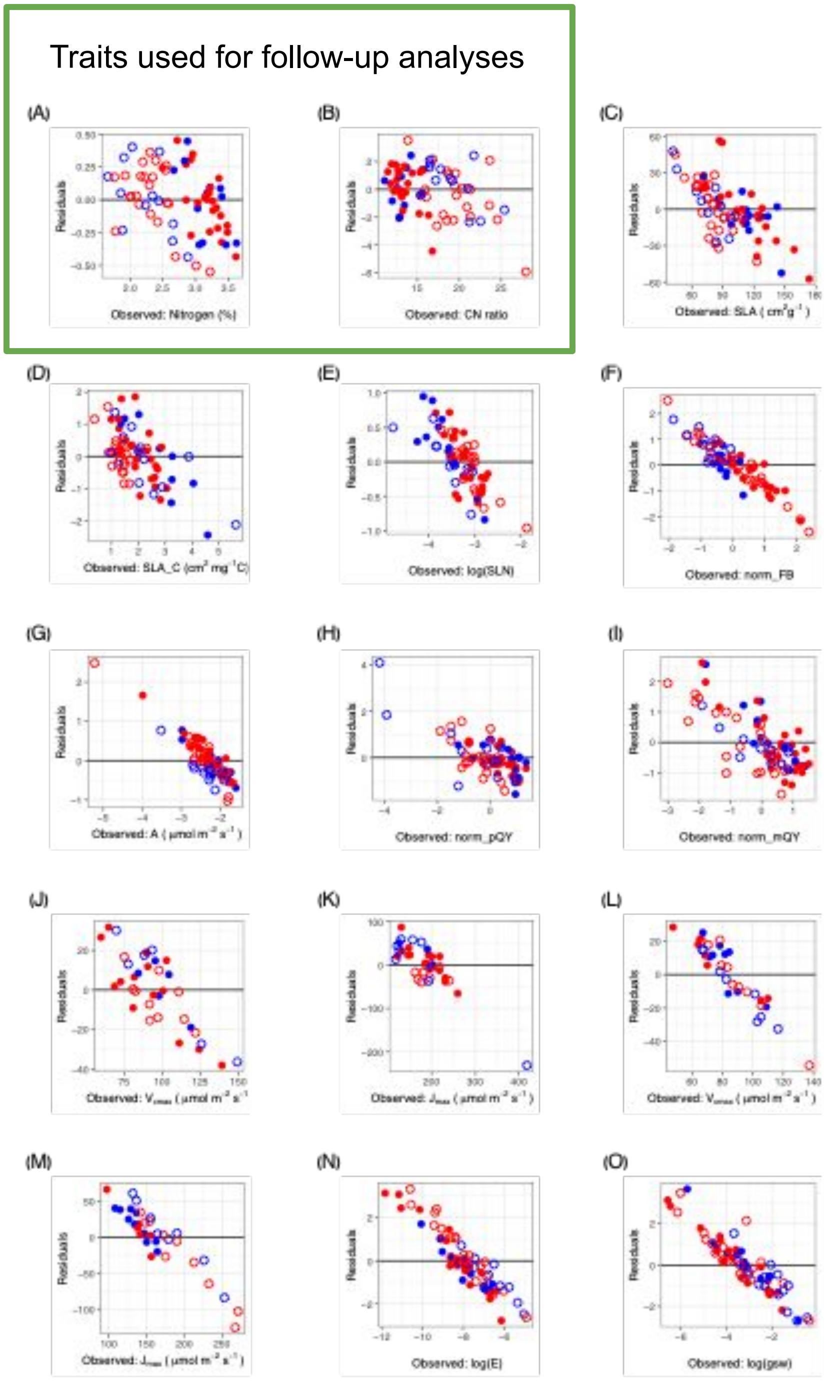

Figure S6. Residuals of calibration models. (A) Nitrogen (N, %) , (B) CN ratio (CN), C) specific leaf area (SLA, cm^2 g^{-1}), (D) specific leaf area with respect to carbon (SLA_C, cm^2 $mg^{-1}C$), (E) log specific leaf nitrogen (log(SLN)), (F) normalized final biomass (norm_FB), (G) pre-dawn leaf-level assimilation (A, µmol m^{-2} s^{-1}), (H) normalized pre-dawn quantum yield (norm_pQY), (I) normalized midday quantum yield (norm_mQY), (J) Vcmax on Week 9 (Vcmax, µmol m^{-2} s^{-1}), (K) Jmax on Week 9 (Jmax, µmol m^{-2} s^{-1}) , (L) Vcmax on Week 13 (Vcmax, µmol m^{-2} s^{-1}), (M) Jmax on Week 13 (Jmax, µmol m^{-2} s^{-1}), (N) log pre-dawn leaf-level transpiration (log(E)), and (O) log pre-dawn leaf-level stomatal conductance to water (log(gsw)). Open circles = N2 treatment; filled circles are N1 treatment; red = IND and blue = TRJ. *Subsequent analyses only focus on N and CN ratio (highlighted in the green box).*

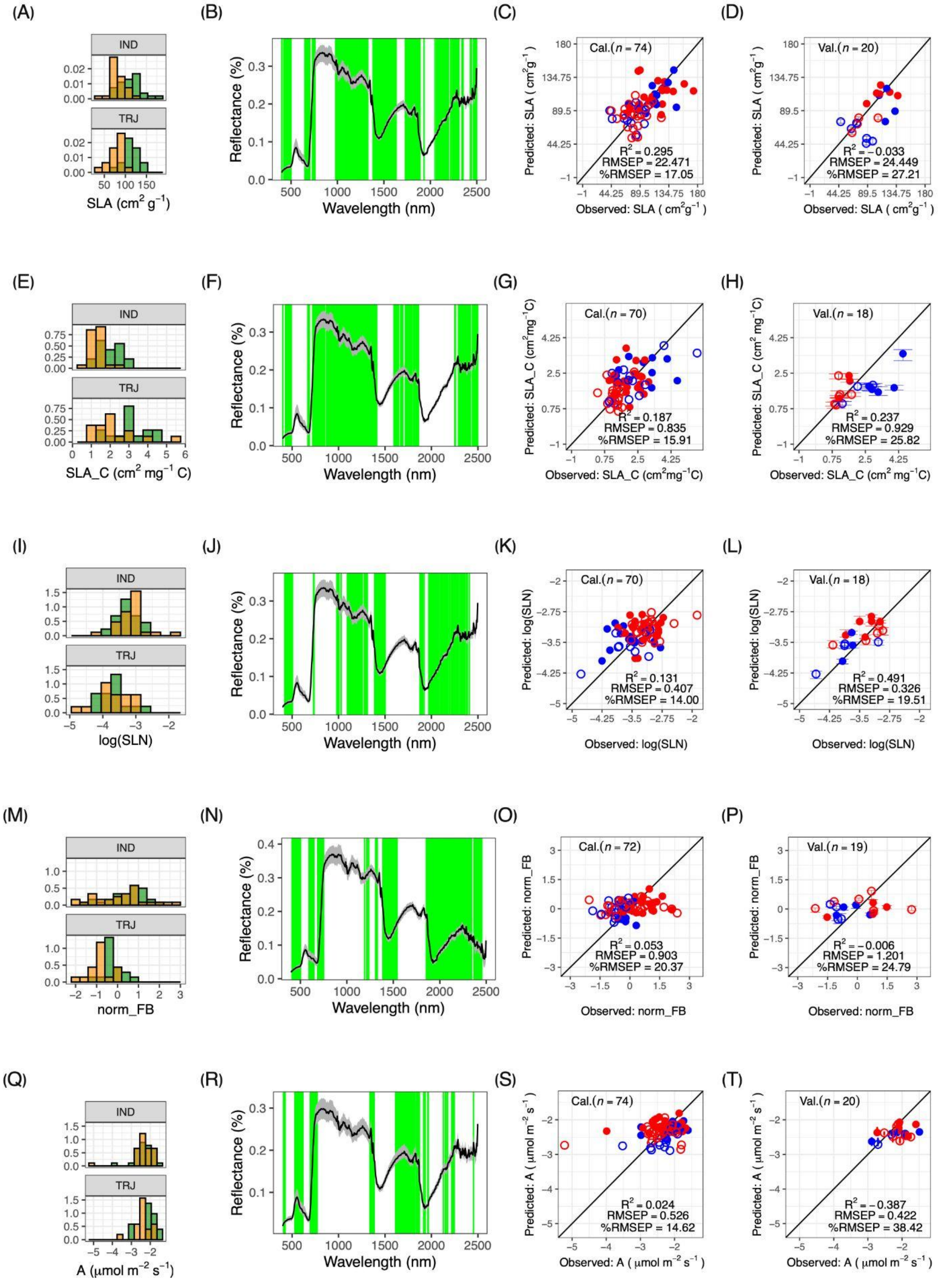

Figure S7. Predictions of traits related to growth. Histograms of each trait are shown in column one. (A) specific leaf area (SLA, $cm^2\ g^{-1}$), (E) specific leaf area with respect to carbon (SLA_C, $cm^2\ mg^{-1}C$), (I) log specific leaf nitrogen (log(SLN)), (M) normalized final biomass (norm_FB), and (Q) pre-dawn leaf-level assimilation (A, $\mu mol\ m^{-2}\ s^{-1}$). Green is high nitrogen treatment (N1) and orange is low nitrogen treatment (N2). Their corresponding selected wavelengths are shown in column two in green. Black lines show mean reflectance values and gray shaded areas indicate reflectances between the 5th and 95th percentiles. Columns three and four are model calibration (C, G, K, O and S) and validation (D, H, L, P and T), respectively. Error bars are 95% confidence intervals. In panels three and four: Open circles = N2 treatment; filled circles = N1 treatment; red = IND and blue = TRJ.

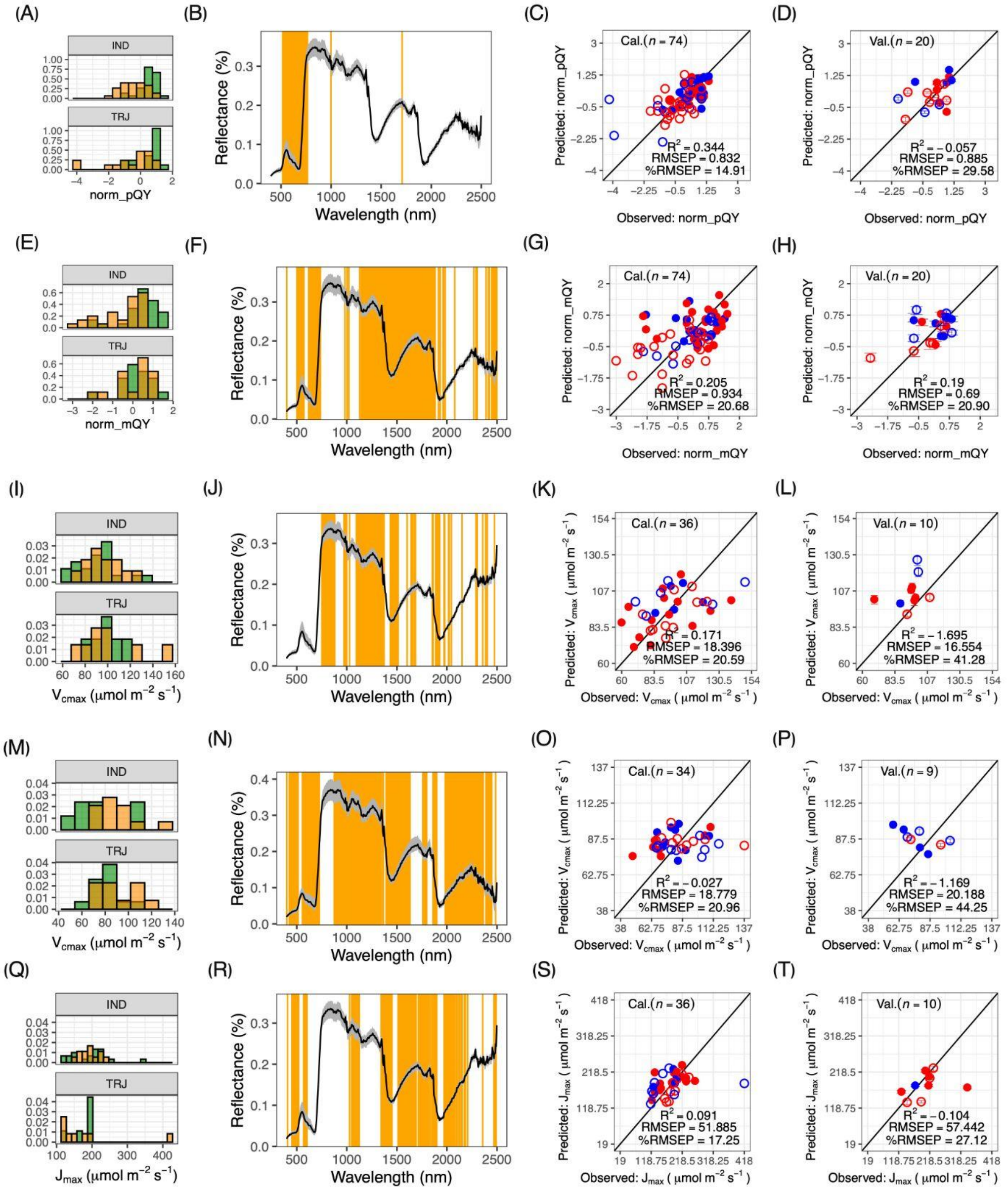

Figure S8. Predictions of traits related to photosynthesis capacity. Histograms of each trait are shown in column one. (A) normalized pre-dawn quantum yield (norm_pQY), (E) normalized midday quantum yield (norm_mQY), (I) Vcmax on Week 9 (Vcmax, μmol m^{-2} s^{-1}), (M) Vcmax on Week 13 (Vcmax, μmol m^{-2} s^{-1}), and (Q) Jmax on Week 9 (Jmax, μmol m^{-2} s^{-1}). Green is high nitrogen treatment (N1) and orange is low nitrogen treatment (N2). (B), (F), (J), (N), and (R) are corresponding selected wavelengths (column two in yellow). Black lines show mean reflectance values and gray shaded areas indicate reflectances between the 5th and 95th percentiles. Columns three and four are model calibration (C, G, K, O, and S) and validation (D, H, L, P, and T), respectively. Error bars are 95% confidence intervals.In panels three and four: Open circles = N2 treatment; filled circles = N1 treatment; red = IND and blue = TRJ.

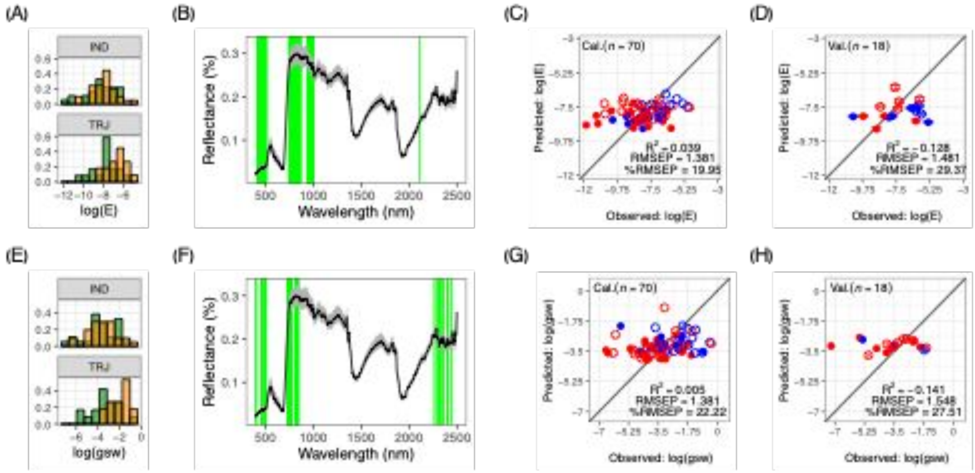

Figure S9. Predictions of traits related to water transport. Histograms of each trait are shown in column one. (A) log pre-dawn leaf-level transpiration (log(E)), and (E) log pre-dawn leaf-level stomatal conductance to water (log(gsw)). Green is high nitrogen treatment (N1) and orange is low nitrogen treatment (N2). (B) and (F) are corresponding selected wavelengths (column two in green). Black lines show mean reflectance values and gray shaded areas indicate reflectances between the 5th and 95th percentiles. Columns three and four are model calibration (C and G) and validation (D and H), respectively. Error bars are 95% confidence intervals. In panels three and four: Open circles = N2 treatment; filled circles = N1 treatment; red = IND and blue = TRJ.

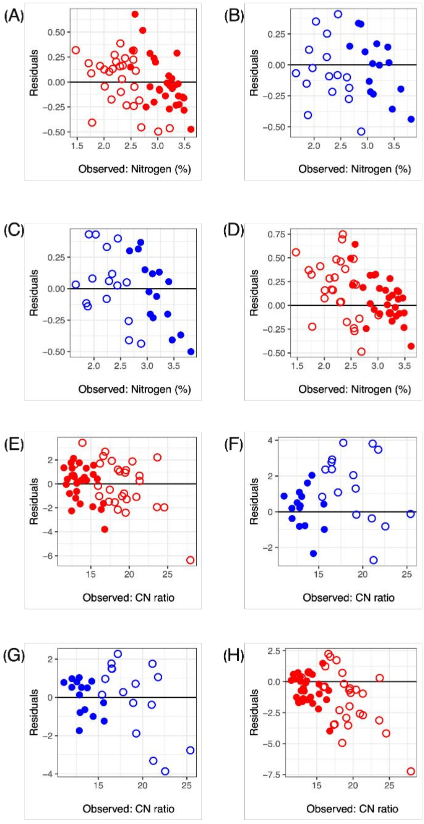

Figure S10. Residuals of calibration and validation for subpopulation-based models for N and CN ratio. (A) and (B) are residuals of the model built on calibrating IND N data and validated on TRJ N data, respectively. (C) and (D) are residuals of the model built on calibrating TRJ N data and validated on IND N data, respectively. (E) and (F) are residuals of the model built on calibrating IND CN ratio data and validated on TRJ CN ratio data, respectively. (G) and (H) are residuals of the model built on calibrating TRJ CN ratio data and validated on IND CN ratio data, respectively. Open circles = N2 treatment; filled circles = N1 treatment; red = IND and blue = TRJ.

Table S1 Germplasm information for the rice panel

Accession[1]	Variety Name	Country	Subpopulation[2]
NSFTV107	NSF-TV 107	Bangladesh	TRJ
NSFTV130	Peh-Kuh-Tsao-Tu	Taiwan	IND
NSFTV137	RTS14	Vietnam	IND
NSFTV17	Binulawan	Philippines	IND
NSFTV189	Criollo La Fria	Venezuela	IND
NSFTV203	Radin Ebos 33	Malaysia	IND
NSFTV222	Paraiba Chines Nova	Brazil	IND
NSFTV223	Priano Guaira	Brazil	TRJ
NSFTV226	IRAT 44	Burkina Faso	TRJ
NSFTV235	Sze Guen Zim	China	IND
NSFTV239	WAB 502-13-4-1	Cote D'Ivoire	TRJ
NSFTV252	Djimoron	Guinea	IND
NSFTV254	Hon Chim	Hong Kong	IND
NSFTV284	IR-44595	Nepal	IND
NSFTV285	Tox 782-20-1	Nigeria	TRJ
NSFTV313	BR24	Bangladesh	IND
NSFTV339	Yodanya	Myanmar	IND
NSFTV385	Nira	United States	IND
NSFTV397	Cybonnet	United States	TRJ
NSFTV59	Gogo Lempuk	Indonesia	TRJ
NSFTV616	RT0034	United States	IND
NSFTV628	Jefferson	United States	TRJ
NSFTV90	Kiang-Chou-Chiu	Taiwan	IND

Note:

1. NSFTV refers to IDs of accessions from Rice Diversity Panel 1.
2. TRJ and IND are *tropical japonica* and *indica* subpopulations, respectively.

Table S2 Timing of physiological trait measurements and imaging dates

Week	Physiological trait	Physiological trait Measurements date (DAS)	Imaging date (DAS)
6	*E*, *A*, *gsw*	39, 40, 41	39, 41
9	*SLA, SLA_C, SLN*, *C*, *N*, *C:N*, A/Ci	59, 60, 61, 63	59, 60, 61, 63
10	*mQY*, *pQY*	67, 69, 71	68, 70
13	A/Ci, *FB*	87, 88, 89, 90, 91, 94[1]	87, 88, 89, 90, 91

Note:

1. *FB* was measured on 94 DAS. Its corresponding imaging date was considered to be from 87 to 91 DAS.
2. *DAS, SLA*, *C*, *N*, *C:N*, *SLA_C*, *SLN*, *FB*, *mQY*, *pQY*, *E*, *A*, *gsw* and A/Ci are days after sowing, specific leaf area ($cm^2\ g^{-1}$), carbon (%), nitrogen (%), CN ratio, specific leaf area with respect to carbon ($cm^2\ mg^{-1}$ C), specific leaf nitrogen (mg N cm^{-2}), final biomass (g), midday quantum yield, pre-dawn quantum yield, pre-dawn leaf-level transpiration (mol $m^{-2}\ s^{-1}$), pre-dawn leaf-level assimilation (μmol $m^{-2}\ s^{-1}$), pre-dawn leaf-level stomatal conductance to water (mol $m^{-2}\ s^{-1}$), and photosynthesis CO_2 response curve, respectively.

Table S3 Summary statistics of physiological traits. Data are mean±sd under high nitrogen treatment (N1) and low nitrogen treatment (N2) for *indica* (IND, 15 genotypes x 2 replicates) and *tropical japonica* (TRJ, 7 genotypes x 2 replicates and cv. Cybonnet has 3 replicates).

Traits	N1		N2	
	IND	TRJ	IND	TRJ
SLA	110.45±25.55	115.05±18.99	80.91±17.02	76.60±19.03
C	42.33±1.92	41.97±2.43	42.72±1.67	42.32±2.36
N	3.12±0.31	3.18±0.31	2.30±0.39	2.26±0.35
C:N	13.71±1.49	13.28±1.27	19.11±3.21	19.13±2.84
SLA_C	1.97±0.61	3.07±0.97	1.28±0.36	2.26±1.18
SLN	0.04±0.012	0.03±0.013	0.05±0.025	0.03±0.012
FB	167.68±24.11	147.83±10.90	160.12±33.36	132.79±13.33
mQY	0.72±0.026	0.71±0.023	0.69±0.033	0.71±0.026
pQY	0.83±0.012	0.81±0.019	0.81±0.019	0.8±0.037
E	0.00049±0.00063	0.00077±0.00113	0.00077±0.00140	0.00192±0.00172
A	-2.3±0.47	-2.11±0.45	-2.28±0.63	-2.36±0.39
gsw	0.05±0.058	0.07±0.101	0.07±0.130	0.17±0.162
V_{cmax} (W9)	91.38±19.72	98.05±12.48	97.14±14.39	100.56±25.81
V_{cmax} (W13)	75.84±23.02	79.17±12.76	88.55±19.26	88.50±17.84
V_{cmax}(W9+W13)	87.35±21.32	85.13±15.27	92.84±17.20	93.58±21.76
J_{max} (W9)	193.28±53.72	180.41±28.69	192.81±28.67	179.74±100.28
J_{max} (W13)	156.86±53.53	141.04±23.52	189.87±45.88	173.97±37.16
J_{max} (W9+W13)	183.84±55.09	153.48±30.84	191.34±37.44	176.40±68.46

Note:
SLA, *C*, *N*, *C:N*, *SLA_C*, *SLN*, *FB*, *mQY*, *pQY*, *E*, *A*, and *gsw* are specific leaf area ($cm^2 g^{-1}$), carbon (%), nitrogen (%), CN ratio, specific leaf area with respect to carbon ($cm^2 mg^{-1}$ C), specific leaf nitrogen (mg N cm^{-2}), final biomass (g), midday quantum yield, pre-dawn quantum yield, pre-dawn leaf-level transpiration (mol $m^{-2} s^{-1}$), pre-dawn leaf-level assimilation (μmol $m^{-2} s^{-1}$) and pre-dawn leaf-level stomatal conductance to water (mol $m^{-2} s^{-1}$), respectively. W9 and W13 are Weeks 9 and 13, respectively.
